# Two-Step Recruitment of RNA-Directed DNA Methylation to Tandem Repeats

**DOI:** 10.1371/journal.pbio.0040363

**Published:** 2006-10-24

**Authors:** Simon W.-L Chan, Xiaoyu Zhang, Yana V Bernatavichute, Steven E Jacobsen

**Affiliations:** 1 Department of Molecular, Cell, and Developmental Biology, University of California Los Angeles, Los Angeles, California, United States of America; 2 Howard Hughes Medical Institute, University of California Los Angeles, Los Angeles, California, United States of America; Max Planck Institute for Developmental Biology, Germany

## Abstract

Tandem repeat sequences are frequently associated with gene silencing phenomena. The *Arabidopsis thaliana FWA* gene contains two tandem repeats and is an efficient target for RNA-directed de novo DNA methylation when it is transformed into plants. We showed that the *FWA* tandem repeats are necessary and sufficient for de novo DNA methylation and that repeated character rather than intrinsic sequence is likely important. Endogenous *FWA* can adopt either of two stable epigenetic states: methylated and silenced or unmethylated and active. Surprisingly, we found small interfering RNAs (siRNAs) associated with *FWA* in both states. Despite this, only the methylated form of endogenous *FWA* could recruit further RNA-directed DNA methylation or cause efficient de novo methylation of transgenic *FWA*. This suggests that RNA-directed DNA methylation occurs in two steps: first, the initial recruitment of the siRNA-producing machinery, and second, siRNA-directed DNA methylation either in *cis* or in *trans*. The efficiency of this second step varies depending on the nature of the siRNA-producing locus, and at some loci, it may require pre-existing chromatin modifications such as DNA methylation itself. Enhancement of RNA-directed DNA methylation by pre-existing DNA methylation could create a self-reinforcing system to enhance the stability of silencing. Tandem repeats throughout the *Arabidopsis* genome produce siRNAs, suggesting that repeat acquisition may be a general mechanism for the evolution of gene silencing.

## Abstract 2

In*Arabidopsis,* methylation and silencing mediated by the*FWA* transgene are found to require the tandem repeat sequences upstream of*FWA* and to be independent of siRNA association.

## Introduction

Cytosine methylation of DNA protects eukaryote genomes from transposons, and also regulates developmental gene expression [1,2]. Tandem repeat sequences are common in methylated regions of the genome. The tandem repeats in retrotransposons are essential for their mobility and are frequently transcriptionally silenced by DNA methylation [3,4]. Tandem repeats upstream of the maize *b1* gene are required for paramutation, a process in which a heritable silent state is transferred from one allele to another [5]. In addition, tandem repeats control imprinting of the *RasGRF1* gene in mammals and are associated with other imprinted genes in mammals and in plants [6–9]. One such example is the *FWA* gene of Arabidopsis thaliana. In adult tissues, *FWA* is silenced by DNA methylation on two pairs of tandem repeats at the 5' end of its transcribed region [10]. The maternal copy of *FWA* is specifically demethylated and expressed in the extra-embryonic endosperm tissue, while paternal *FWA* remains methylated and silent [6]. Because *FWA* is only demethylated in the terminally differentiating endosperm, it remains heritably methylated throughout the plant life cycle. Rare unmethylated epigenetic alleles of *FWA* (termed *fwa*) ectopically overexpress the FWA transcription factor in adult tissues, causing a dominant late-flowering phenotype [10]. These alleles are very stable, and remethylation that re-establishes an early-flowering phenotype has never been observed.

In both plants and animals, de novo DNA methylation of previously unmethylated sequences is mediated by DNA methyltransferase enzymes of the Dnmt3 family (DOMAINS REARRANGED METHYLTRANSFERASE2 [DRM2] in *Arabidopsis*) [11]. DRM2 is responsible for de novo DNA methylation in all known sequence contexts: CG, CNG (where N is any base), and asymmetric or CHH (where H = A, T, or C) [11]. However, the maintenance of pre-existing DNA methylation is sequence context–dependent. CG DNA methylation is maintained by MET1 (a homolog of mammalian Dnmt1), whereas CNG and asymmetric methylation are maintained by the overlapping functions of CHROMOMETHYLASE3 (CMT3) and DRM2 [2,12].

De novo DNA methylation can be studied in *Arabidopsis* by transforming plants with an additional copy of *FWA* [11]. Despite the extreme stability of an unmethylated *fwa* endogenous allele, a transgenic copy of *FWA* introduced into wild-type plants by *Agrobacterium*-mediated transformation is always de novo methylated and silenced. Because both endogenous and transgenic *FWA* are silenced, transformed plants do not display a late-flowering phenotype. However, when *drm2* mutant plants are transformed with *FWA,* the introduced transgenes remain unmethylated, and *FWA* overexpression causes a late-flowering phenotype. In *drm2* transformants, endogenous *FWA* retains its methylation and silencing, because DRM2 only controls de novo methylation but not maintenance methylation of CG sites [11]. Intriguingly, when unmethylated *FWA* transgenes from *drm2* transformants are outcrossed to reintroduce wild-type *DRM2,* the transgenes remain stably unmethylated [11]. This is consistent with the stability of unmethylated *fwa* endogenous alleles and suggests that *FWA* de novo DNA methylation only occurs during the process of plant transformation.

RNA interference (RNAi) is a versatile silencing pathway in which double-stranded RNA is processed by Dicer nucleases to generate 21–24-nucleotide small interfering RNAs (siRNAs) [13]. siRNAs can down-regulate gene expression through mRNA cleavage, translational inhibition, or transcriptional silencing. Previously, we used the *FWA* transformation assay to demonstrate that de novo DNA methylation by DRM2 is guided by an RNAi pathway including the NUCLEAR RNA POLYMERASE IV A (NRPD1a) subunit of DNA-dependent RNA polymerase IV (RNA Pol IV), RNA-DEPENDENT RNA POLYMERASE2 (RDR2), DICER-LIKE3 (DCL3), and ARGONAUTE4 (AGO4) [14]. The recent discovery that a maize ortholog of *RDR2* is required for paramutation demonstrates that RNA-directed DNA methylation at tandem repeats is conserved in both monocots and eudicots [15]. In addition to their defects in establishment of DNA methylation, *Arabidopsis* RNAi mutants reduce or eliminate CNG and asymmetric DNA methylation at endogenous tandem repeats and other sequences in a manner analogous to that of *drm2* mutants. However, at most loci, the maintenance of CG methylation in *drm2* or the RNAi mutants is generally unaffected. These results show that RNAi-related processes are required to propagate non-CG DNA methylation; meaning that, even after establishment, siRNA production likely persists at particular genomic loci.

Although it seems clear that de novo DNA methylation derives its sequence specificity from siRNAs, it is not known why transformed *FWA* is targeted for DNA methylation, unlike the majority of single-copy *Arabidopsis* genes transformed into plants. Here we show that the tandem repeats of *FWA* are necessary and sufficient for triggering de novo DNA methylation, and that repeated character (rather than the *FWA* sequence itself) is likely critical. Transformed *FWA* can silence the unmethylated endogenous *fwa* gene in *trans,* and this property also requires tandem repeats. Surprisingly, the methylation state of endogenous *FWA* can affect silencing of an incoming transgene, because *FWA* transformed into *fwa-1* is inefficiently methylated compared to wild type. The fact that DNA methylation enhances the ability of endogenous *FWA* to communicate with transformed *FWA* is particularly striking, because both methylated and unmethylated forms of endogenous *FWA* produce equivalent levels of siRNAs. Furthermore, we show that *FWA* siRNA synthesis is dependent on wild-type NRPD1a, RDR2, and DCL3, but not on the putatively downstream components NRPD1b, AGO4, DEFECTIVE IN RNA-DIRECTED DNA METHYLATION1 (DRD1), and DRM2. Our results suggest a two-step model for DNA methylation, in which tandem repeats are sufficient to recruit NRPD1a, RDR2, and DCL3 to produce siRNAs, but a second triggering event such as plant transformation is required to allow siRNAs to target downstream DNA methylation. Finally, a genomic analysis suggests that unique tandem repeats, such as those at *FWA,* are common targets for the siRNA machinery, indicating the potential generality of this gene silencing mechanism on an evolutionary time scale.

## Results

### *FWA* De Novo DNA Methylation during Plant Transformation Can Occur after Fertilization of the Female Gametophyte

**Figure 1:**
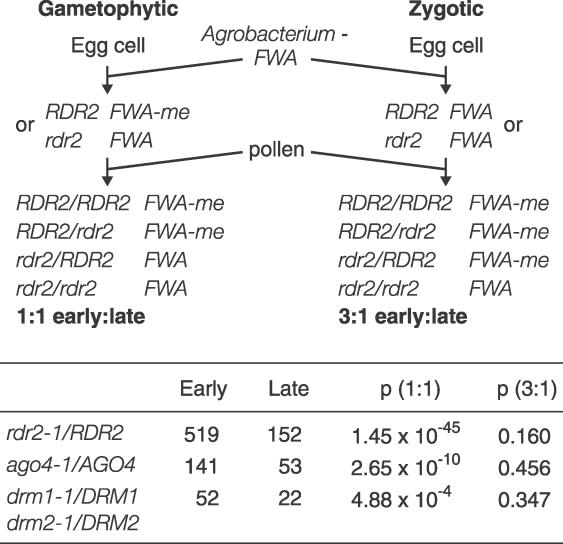
De Novo DNA Methylation of Transformed *FWA* Can Be Rescued by Wild-Type Genes from Pollen Heterozygous *rdr2–1, ago4–1,* and *drm1–1 drm2–1* plants were transformed with *FWA*. If the gametophytic genotype controls de novo DNA methylation and silencing, a 1:1 ratio of early- to late-flowering plants is predicted. If a wild-type gene from pollen can rescue de novo DNA methylation in the fertilized zygote, a 3:1 early- to late-flowering ratio results. The ratio of early- to late-flowering plants was determined in the T1 generation (see Figure S1 for determination of early versus late flowering in each ecotype). The probability of the observed results occurring by chance assuming a 1:1 or 3:1 ratio was calculated with the χ^2^ test.

Although an unmethylated *fwa* endogenous allele never becomes spontaneously methylated, de novo DNA methylation of transformed *FWA* occurs with 100% efficiency [11,14]. Furthermore, unmethylated *FWA* transgenes in *drm2* transformants are not de novo methylated when crossed to plants containing functional *DRM2*. This suggests that de novo methylation of *FWA* occurs either during the transformation process, or shortly thereafter, and we sought to determine more precisely when this event occurs. During the floral dip procedure, Agrobacterium tumefaciens transforms the female gametophytic lineage of *Arabidopsis* (probably the egg cell itself), integrating its transferred DNA (T-DNA) only into the maternal genome [16,17]. We transformed *FWA* into heterozygous *rdr2–1/RDR2, ago4–1/AGO4,* and *drm1–1/DRM1 drm2–1/DRM2* plants (Figure 1). If DNA methylation and gene silencing must be established in the female gametophyte, then the haploid genotype of the gametophyte should control de novo DNA methylation and one-half of the transformed T1 progeny would be late flowering. However, if a wild-type *RDR2, AGO4,* or *DRM2* gene from the pollen could rescue de novo DNA methylation in the fertilized zygote, only one in four plants would fail to silence *FWA* and flower late (Figure 1; this ratio could also be explained if de novo DNA methylation requires a factor that moves from the fertilized extra-embryonic endosperm into the zygote). A third possibility is that maternally supplied protein or mRNA could rescue de novo DNA methylation, causing all progeny of a heterozygous mutant to be early flowering.

We found that all three experiments showed an early:late flowering ratio close to 3:1 amongst *FWA* T1 transformants (Figure 1). This early-to-late ratio of flowering plants is consistent with zygotic control, but it could also arise if de novo DNA methylation occurred in the gamete but half the mutant gametes were rescued by maternally supplied protein or mRNA. To eliminate this possibility, we used PCR-based molecular markers to genotype 23 late-flowering T1 progeny of *rdr2–1/RDR2* plants and 18 late-flowering T1 progeny of *ago4–1/AGO4* plants. All were homozygous mutant for *rdr2–1* or *ago4–1,* respectively (unpublished data), indicating that the genotype of the fertilized zygote or endosperm determines whether an *FWA* transgene is silenced. Thus, although *FWA* de novo DNA methylation requires plant transformation, the particular conditions that facilitate de novo DNA methylation must persist until sometime after fertilization.

### *FWA* Tandem Repeats Are Necessary and Sufficient to Trigger De Novo Methylation

DNA methylation of *FWA* is restricted to the two pairs of tandem repeats at the farthest 5' end of its transcribed region, suggesting that they play a key role in controlling gene silencing [10]. The *FWA* tandem repeats are 2 × 38 base pairs (bp) and 2 × 198 bp in length. To test the role of tandem repeats in *FWA* de novo DNA methylation, we cloned several deletion variants of *FWA*. Because some of these variants did not contain the *FWA* open reading frame, they would not be expected to cause late flowering when transformed into wild-type *Arabidopsis,* meaning that we could not use transgene-induced late flowering as an assay. Therefore, we took advantage of the observation that transforming the late-flowering *fwa-1* mutant with a cosmid containing *FWA* causes the occasional appearance of early-flowering plants among first generation T1 transformants [10]. Despite the fact that *fwa-1* plants never regain DNA methylation during normal plant growth, both transgenic and endogenous *FWA* genes contain DNA methylation in early-flowering *fwa-1 + FWA* plants [10]. This shows that transformed *FWA* can target de novo DNA methylation to the unmethylated endogenous *fwa* gene, albeit inefficiently.

**Figure 2:**
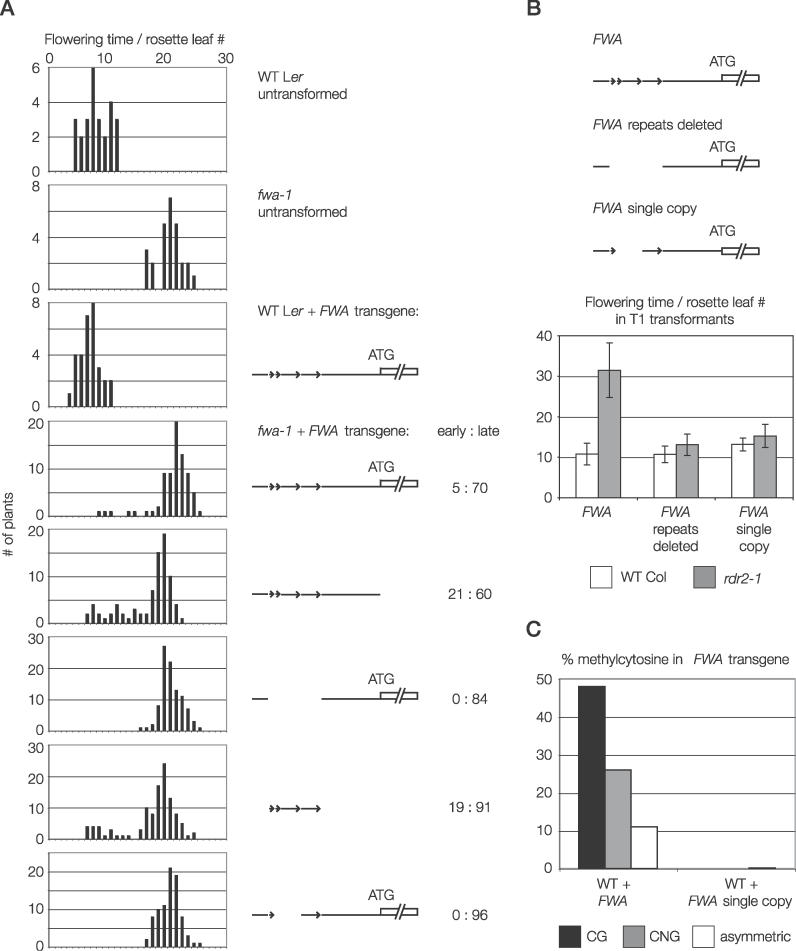
Recruitment of RNA-Directed DNA Methylation to Transformed Direct Repeats (A) Silencing of endogenous *FWA* by transformed *FWA* requires tandem repeats in the incoming transgene. Wild-type (WT) L*er* or *fwa-1* plants were transformed with a complete *FWA* transgene or the deletion variants shown at right. *FWA* contains two tandem repeats, which are depicted as two sets of arrows. Parental *fwa-1* plants are uniformly late flowering. Early flowering in *fwa-1* transformants occurs when endogenous *fwa* is silenced by de novo DNA methylation. Plants were scored as early- (17 leaves or fewer) or late-flowering (18 leaves or more) by comparison to untransformed L*er* and *fwa-1*. (B) *FWA* transgenes lacking tandem repeats do not cause late flowering when transformed into *rdr2–1*. The full *FWA* gene, “*FWA* repeats deleted” transgene, and “*FWA* single copy” transgenes are diagrammed. Wild-type Col and *rdr2–1* plants were transformed with each transgene, and flowering time was assayed in the T1 generation. (C) De novo DNA methylation of transformed *FWA* requires tandem repeat character. Wild-type Col plants were transformed with full-length *FWA* or with single-copy *FWA*. DNA methylation of the *FWA* transgene was assayed by bisulfite sequencing.

We transformed *fwa-1* plants with a 6.1 kilobase *FWA* transgene, and found that 5%–25% of transformants had an early-flowering phenotype (Figure 2A), an effect similar to that caused by *FWA* cosmid transformation. Deleting the *FWA* open reading frame did not abolish the ability of the transgene to silence endogenous *FWA,* but a version lacking the tandem repeats altogether yielded no early-flowering transformants. The tandem repeats alone cloned into an *Agrobacterium* vector were able to cause *trans-*silencing, showing that this region of *FWA* is responsible for the activity. Most importantly, an *FWA* transgene containing a single copy of each tandem repeat sequence (“single-copy *FWA*”) was unable to silence endogenous *fwa*. The “repeats deleted” and “single-copy” transgenes did not cause late flowering when transformed into the *rdr2–1* mutant (Figure 2B), indicating that they are not transcribed even when de novo DNA methylation is compromised. Therefore, the flowering time of *fwa-1* plants that were transformed with these transgenes is not influenced by transgene expression, and transgenes lacking tandem repeat character are incapable of silencing endogenous *FWA*.

It is possible that transcriptional activity of an *FWA* transgene contributes to the silencing of the endogenous copy of *FWA* in *trans*. Therefore, we sought to test de novo DNA methylation of the transgene directly by transforming single-copy *FWA* into wild-type plants and measuring DNA methylation of the transgene by bisulfite genomic sequencing [10]. The complete *FWA* gene transformed into wild type was robustly methylated in the T1 generation, but a single-copy *FWA* transgene was completely unmethylated (Figure 2C). We showed previously that de novo DNA methylation of *FWA* requires the siRNA-metabolizing factors PolIVa, RDR2, DCL3, and AGO4. Together with the data presented here, these results suggest that RNA-directed DNA methylation is recruited to the *FWA* tandem repeat sequences during plant transformation. Although tandem repeats are associated with many gene-silencing phenomena, in this instance it is clear that a single copy of the same sequence lacks the behavior of the tandem repeat. We interpret these results to mean that the repeated character of transformed *FWA,* rather than its intrinsic sequence, is required to target DNA methylation to the unmethylated endogenous gene. An alternate explanation is that a sequence-specific factor recognizes transformed *FWA* and binds cooperatively in a manner that requires two copies of the repeats.

### Efficient Silencing of Transformed *FWA* Requires DNA Methylation of Endogenous *FWA*

**Figure 3:**
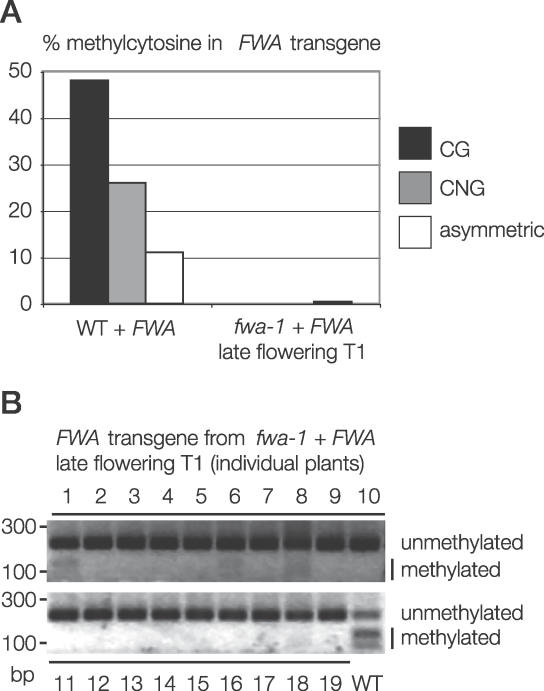
Communication between Methylated Endogenous *FWA* and Transformed *FWA* (A) Efficient de novo DNA methylation of transgenic *FWA* requires DNA methylation at endogenous *FWA*. DNA methylation of an *FWA* transgene introduced into wild-type or *fwa-1* plants was assayed by bisulfite sequencing. For *fwa-1* + *FWA*, three clones were sequenced from each of eight late-flowering individuals (each transformant had >20 rosette leaves at bolting). Early-flowering *fwa-1* transformants were discarded, because they have silenced the *FWA* transgene by de novo DNA methylation. (B) Transgenic *FWA* is unmethylated in many late-flowering *fwa-1* + *FWA* T1 individuals. Before bisulfite treatment, genomic DNA from late-flowering *fwa + FWA* T1 plants was digested with BglII to destroy the endogenous *FWA* gene. DNA methylation of transgenic *FWA* was assayed by PCR from bisulfite-treated DNA followed by ClaI digestion. CG DNA methylation protects the ClaI site from bisulfite conversion, allowing restriction digestion after bisulfite treatment. Wild-type DNA (not digested with BglII) was assayed as a control.

Silencing of unmethylated, endogenous *fwa* by transformed *FWA* shows that an incoming transgene can communicate with an identical endogenous sequence. When unmethylated *fwa-1* mutants are transformed with *FWA,* early-flowering transformants must have silenced both the transgenic and endogenous *FWA* genes, because overexpression of any copy of *FWA* causes a dominant late-flowering phenotype. We sought to determine the fate of the *FWA* transgene in the majority of late-flowering *fwa-1 + FWA* transformants, where endogenous *FWA* is still overexpressed. We expected that the *FWA* transgene would be efficiently methylated in all transformants, reasoning that *cis* methylation of the transgene would be more efficient than *trans* methylation of the endogenous gene induced by the transgene (overexpression of the unmethylated endogenous gene would still cause late flowering, masking silencing of the transgenic copy). To our surprise, however, we found that transgenic *FWA* was completely unmethylated in late-flowering *fwa-1* + *FWA* T1 transformants, when assayed either by bisulfite genomic sequencing (Figure 3A), or by testing 19 individual transformants using a bisulfite PCR/restriction enzyme assay (Figure 3B). *FWA* transformed into wild-type plants is methylated with 100% efficiency, yet our experiment shows that *FWA* transformed into *fwa-1* plants is seldom methylated. Thus, efficient de novo DNA methylation of transformed *FWA* requires DNA methylation of endogenous *FWA.* This demonstrates a second type of communication, in which the endogenous methylated *FWA* gene assists DNA methylation of an incoming transgenic copy.

### siRNAs Are Produced from Both Methylated and Unmethylated Forms of *FWA*

**Figure 4:**
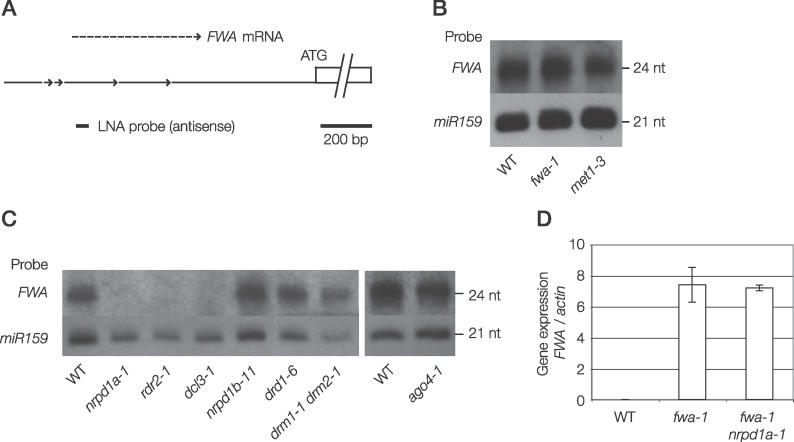
Characterization of *FWA* siRNAs in Different Genetic Backgrounds (A) Schematic diagram of the *FWA* locus and position of LNA probe used to detect siRNAs. (B) *FWA* siRNAs are still produced when the gene is unmethylated. siRNAs from wild type, *fwa-1,* and *met1–3* were analyzed by Northern blotting. miR159 was probed as a loading control. (C) *FWA* siRNAs from plants with the indicated genotype were analyzed by Northern blotting. (D) siRNAs in *fwa-1* do not cause mRNA destruction. The ratio of *FWA* to *ACTIN7* mRNA levels in rosette leaf tissue was measured by RT-qPCR. Reverse transcription was performed with a poly-T oligonucleotide. There was no PCR amplification when reverse transcriptase was omitted (unpublished data).

We wished to correlate these gene silencing phenomena with the production of *FWA* siRNAs. Massively parallel signature sequencing of *Arabidopsis* small RNA has shown that *FWA* siRNAs have very low abundance [18]. Only four siRNAs matching the *FWA* tandem repeats were cloned. Their abundance measured in transcripts per quarter million (TPQ) is 18 in libraries made from floral tissue, in contrast to microRNA 167, which has a TPQ of 59951 in the same libraries [18]. Two previous studies have used Northern blot analysis to detect *FWA* siRNAs using conventional RNA probes [4,19]. However, the *FWA* siRNA signal in these previous experiments was very close to background levels, making it difficult to accurately assess the abundance of *FWA* siRNAs in different genetic backgrounds. To circumvent these problems, we synthesized a locked nucleic acid (LNA) oligonucleotide probe to *FWA* [20], with an LNA backbone substitution at every third position (Figure 4A). Using this strategy, we were able to detect a robust signal for *FWA* 24-nucleotide siRNAs (Figure 4B).

We initially hypothesized that differences in siRNA production might explain why methylated *FWA* in wild-type cells can enhance de novo methylation of transformed *FWA,* whereas unmethylated endogenous *fwa* does not. However, instead we found that *fwa-1* plants contain *FWA* 24-nucleotide siRNAs at levels comparable to wild type (Figure 4B). We confirmed the existence of *FWA* siRNAs that depend on *RDR2* and are present in *fwa-1* by using two additional LNA probes from nonoverlapping regions of the tandem repeats (Figure S2). As further confirmation that siRNAs are produced from the unmethylated *FWA* gene, we also measured *FWA* siRNAs in a *met1–3* DNA methyltransferase mutant that had lost all *FWA* DNA methylation and displayed a late-flowering phenotype [10,21]. Although it was previously reported using conventional probes that *FWA* siRNAs were reduced in *met1* [4], we found using LNA probes that similar levels of *FWA* siRNAs were detected in *met1* and wild-type strains (Figure 4B). Our observation that siRNAs are produced from both unmethylated *fwa* and methylated *FWA* is interesting, because only plants containing a methylated *FWA* endogene can efficiently de novo methylate an incoming transgene. One possibility is that siRNAs produced at methylated *FWA* are qualitatively different from those at unmethylated *fwa*. For example, they might be associated with different downstream effector complexes.

### *FWA* siRNA Production Relies on only a Subset of Factors Required for *FWA* De Novo Methylation

To gain further insight into siRNA production at tandem repeats, we tested for the presence of siRNAs in a battery of mutants that are defective for *FWA* de novo methylation. We previously showed that mutations in four RNA silencing genes—*NRPD1a, RDR2, DCL3,* and *AGO4*—phenocopy the *FWA* de novo methylation defect of the DNA methyltransferase mutant *drm2* [14]. We have more recently found that mutations in two other genes share this phenotype. The first is *NRPD1b*. DNA-dependent RNA polymerase IV exists in two forms in *Arabidopsis* [19,22–24]. Both share the small subunit encoded by *NRPD2a* but have different large subunits encoded by either *NRPD1a* or by *NRPD1b*. We found that, similar to the other RNA silencing mutants, an *nrpd1b-11* T-DNA mutation blocks de novo methylation and silencing of transformed *FWA* [25]. The second new gene involved in *FWA* de novo DNA methylation is *DRD1,* which was isolated in a screen for mutants that lack RNA-directed DNA methylation triggered by a transgenic inverted repeat, and encodes a SNF2-like putative chromatin remodeling protein [26]. The *drd1–6* allele [26] again blocked *FWA* de novo methylation and silencing [27].

We observed that *FWA* siRNA hybridization signal was absent in *nrpd1a-1, rdr2–1,* and *dcl3–1* (Figure 4C). However, we observed 24-nucleotide *FWA* siRNA signals similar to wild-type levels in the *nrpd1b-11, ago4–1, drd1–6,* and *drm1–1 drm2–1* mutants, all of which are defective for *FWA* de novo DNA methylation (Figure 4C). These data suggest that NRPD1a, RDR2 and DCL3 act in the initial formation of siRNAs, whereas NRPD1b, AGO4, DRD1, and DRM2 may act at more downstream steps of RNA-directed DNA methylation. Our finding that *FWA* siRNAs are stable in the absence of AGO4 is particularly interesting, because ARGONAUTE proteins are the siRNA binding moieties in RNAi pathways [13]. It is possible that other *Arabidopsis* ARGONAUTE proteins bind to *FWA* siRNAs in *ago4–1* mutant plants, preserving their stability (the *Arabidopsis* genome encodes ten ARGONAUTE proteins). If this is the case, however, these alternative ARGONAUTEs are not capable of replacing the function of AGO4 in RNA-directed DNA methylation. The observation that NRPD1a is required for *FWA* siRNA production, yet NRPD1b is not, supports the conclusion of Kanno et al. and Pontier et al. that the two forms of RNA Pol IV play distinct roles in the process of RNA-directed DNA methylation [19,23]. The behavior of *FWA* siRNAs contrasts with that of siRNAs from the small euchromatic SINE transposon *AtSN1,* the levels of which are greatly reduced in *ago4, nrpd1b,* and *drm1 drm2* mutants [19,28,29]; *AtSN1* may be a locus where feedback from downstream DNA methylation is required to maintain siRNA production.

**Figure 5:**
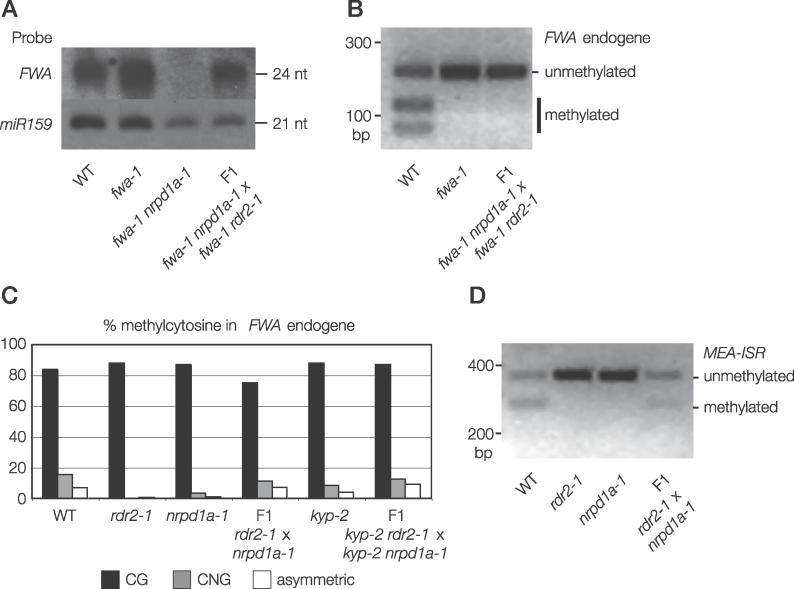
Separable Recruitment of siRNA Production and Non-CG DNA Methylation at Chromosomal Tandem Repeats (A) Unmethylated chromosomal tandem repeats can recruit siRNA production. *fwa-1 nrpd1a-1* and *fwa-1 rdr2–1* lack *FWA* siRNAs, but when these mutants are crossed together, Northern blotting shows that *FWA* siRNA production is restored in F1 plants. (B) Unmethylated chromosomal tandem repeats cannot recruit de novo DNA methylation. When *fwa-1 nrpd1a-1* and *fwa-1 rdr2–1* mutants are crossed together, *FWA* remains unmethylated despite resumption of siRNA production. DNA methylation of *FWA* was assayed by PCR from bisulfite-treated DNA followed by ClaI digestion. (C) CG DNA methylation recruits siRNA-directed non-CG DNA methylation to *FWA*. When the recessive *rdr2–1* and *nrpd1a-1* mutants are crossed together, non-CG DNA methylation returns to CG-methylated *FWA* in the F1 plants. DNA methylation was assayed by bisulfite sequencing. (D) CG DNA methylation recruits siRNA-directed non-CG DNA methylation to *MEA-ISR*. Non-CG DNA methylation returns to CG-methylated *MEA-ISR* in *rdr2–1* × *nrpd1a-1* F1 plants. Asymmetric DNA methylation was assayed by PCR from bisulfite-treated DNA followed by BamHI digestion. DNA methylation protects the BamHI site from bisulfite conversion, allowing restriction digestion after bisulfite treatment.

It is curious that NRPD1a/RDR2/DCL3–dependent *FWA* siRNAs are produced from unmethylated *fwa* alleles and yet do not cause DNA methylation. This prompted us to ask if these siRNAs instead might be causing post-transcriptional mRNA destruction. To test this, we used reverse transcription coupled to real-time quantitative PCR (RT-qPCR) to compare the levels of mRNA produced in either *fwa-1* plants that produce *FWA* siRNAs or in *fwa-1 nrpd1a-1* plants, where *FWA* siRNAs are undetectable (Figures 4D and 5A). *FWA* mRNA was present at equivalent levels in *fwa-1* and *fwa-1 nrpd1a-1,* showing that the siRNAs present in *fwa-1* are not diverted into a post-transcriptional gene-silencing pathway if DNA methylation is absent. Thus the function of *FWA* siRNAs in the unmethylated *fwa-1* mutant remains obscure. Other endogenous *Arabidopsis* siRNAs match regions of the genome not known to contain DNA methylation [30], and the function of these are also unknown.

### Unmethylated Tandem Repeats Can Recruit siRNA Production without Downstream De Novo DNA Methylation

During *Agrobacterium-*mediated transformation, tandem repeats are recognized by siRNA-producing factors that guide DNA methylation, yet our *fwa-1* results showed that an unmethylated gene can also produce siRNAs. To test whether unmethylated tandem repeats can recruit siRNA production independent of plant transformation, we first constructed two double mutants, *fwa-1 nrpd1a-1* and *fwa-1 rdr2–1,* which lack DNA methylation at *FWA* and are also defective for siRNA production. We then crossed these plants together to reintroduce wild-type *NRPD1a* and *RDR2*. In the F1 of this cross, we observed that siRNA production at *FWA* immediately resumed (Figure 5A). This result shows directly that the *FWA* tandem repeats in their native chromosomal context can recruit Pol IVa, RDR2 and DCL3 activity to produce siRNAs independently of the DNA transport and integration steps of plant transformation. Interestingly, the *fwa* gene remained unmethylated in the F1 of the *fwa-1 nrpd1a-1* × *fwa-1 rdr2–1* cross (Figure 5B). This result shows that *FWA* siRNAs cannot cause de novo DNA methylation of unmethylated *fwa* in its native genomic context, even when their production is initiated afresh as it must be at a transformed copy of *FWA*.

### Tandem Repeats with Pre-Existing CG DNA Methylation Recruit Both siRNA Production and RNA-Directed Non-CG Methylation

The inability of *FWA* siRNAs to de novo DNA methylate the *fwa* endogene can be contrasted with methylated *FWA,* where siRNAs persistently target non-CG DNA methylation. One feature of methylated *FWA* that may facilitate siRNA-directed DNA methylation is CG DNA methylation, which does not require siRNAs for its inheritance. We tested whether recruitment of siRNA production to CG-methylated *FWA* could target non-CG DNA methylation by crossing together homozygous *nrpd1a-1* and *rdr2–1* mutants, thereby reintroducing wild-type *NRPD1A* and *RDR2*. In *nrpd1a* and *rdr2* (like in *drm2*), tandem repeats at *FWA* and *MEDEA INTERGENIC SUBTELOMERIC REPEAT (MEA-ISR)* lose their non-CG DNA methylation but fully retain CG methylation [14]. *FWA* remains silent in these plants (which have an early-flowering phenotype), because CG methylation is sufficient to suppress *FWA* expression. In *nrpd1a-1* × *rdr2–1* F1 plants, non-CG DNA methylation was immediately restored at both *FWA* and *MEA-ISR* (Figure 5C and 5D). In comparison, unmethylated *fwa* never spontaneously regains DNA methylation. These data suggest that CG methylation, or another chromatin modification that is associated with CG methylation, can recruit RNA silencing effector complexes and DRM2 to methylate non-CG sites. The fact that *MEA-ISR* behaves like *FWA* is important because it illustrates that recruitment of RNA-directed DNA methylation to tandem repeats is not a specialized property of a single gene.

Histone H3 lysine 9 dimethylation (H3K9me2) is a post-translational modification associated with gene silencing and has been shown to control non-CG DNA methylation in *Arabidopsis* [31,32]. Furthermore, H3K9me2 acts downstream of CG DNA methylation, because the majority of H3K9me2 is lost in a *met1* mutant [33]. We therefore sought to test whether H3K9me2 might be involved in the recruitment of siRNA-directed non-CG methylation factors to CG methylated tandem repeats. The majority of H3K9me2 in *Arabidopsis* is maintained by the SET domain protein KRYPTONITE (KYP)/SUVH4, and the *kyp-2* mutation strongly reduces H3K9me2 at the *FWA* tandem repeats [34]. We constructed double mutants of *kyp-2 nrpd1a-1* and *kyp-2 rdr2–1,* then crossed these plants together to reintroduce wild-type *NRPD1a* and *RDR2* in a background that is still homozygous for *kyp-2*. In the F1 of the *kyp-2 nrpd1a-1* × *kyp-2 rdr2–1* cross, asymmetric DNA methylation at *FWA* returned to the same levels as *kyp-2* (Figure 5C). Thus, tandem repeats at *FWA* that have CG DNA methylation but lack KYP-dependent H3K9me2 are still capable of recruiting RNA-directed non-CG DNA methylation.

### Tandem Repeat Sequences throughout the *Arabidopsis* Genome Are Enriched for siRNAs

Our results using *FWA* as a model provide evidence that tandem repeats recruit RNA silencing proteins to produce siRNAs. To assess the generality of these findings, we correlated tandem repeat sequences throughout the *Arabidopsis* genome with a large database of siRNAs cloned by massively parallel signature sequencing [18]. We used the program Tandem Repeats Finder [35] to define each tandem repeat in the genome with a repeat unit length greater than 20 bp (to avoid microsatellites). Tandem repeats that are found in multiple genomic locations may resemble transposons in that they could be sensed by a cellular mechanism devoted to high-copy dispersed repeats. We therefore filtered our tandem repeat library to remove any type of dispersed or inverted repeat character. We used BLAST (set to detect short, nearly perfect matches) to filter tandem repeats present at more than one genomic locus, RepBase to remove additional annotated transposons and repeats, and Inverted Repeats Finder [36] to exclude inverted repeats. Lastly, we restricted our analysis to tandem repeats outside of pericentromeric heterochromatin, because these should be less susceptible to silencing by adjacent transposons and high-copy sequences.

**Figure 6:**
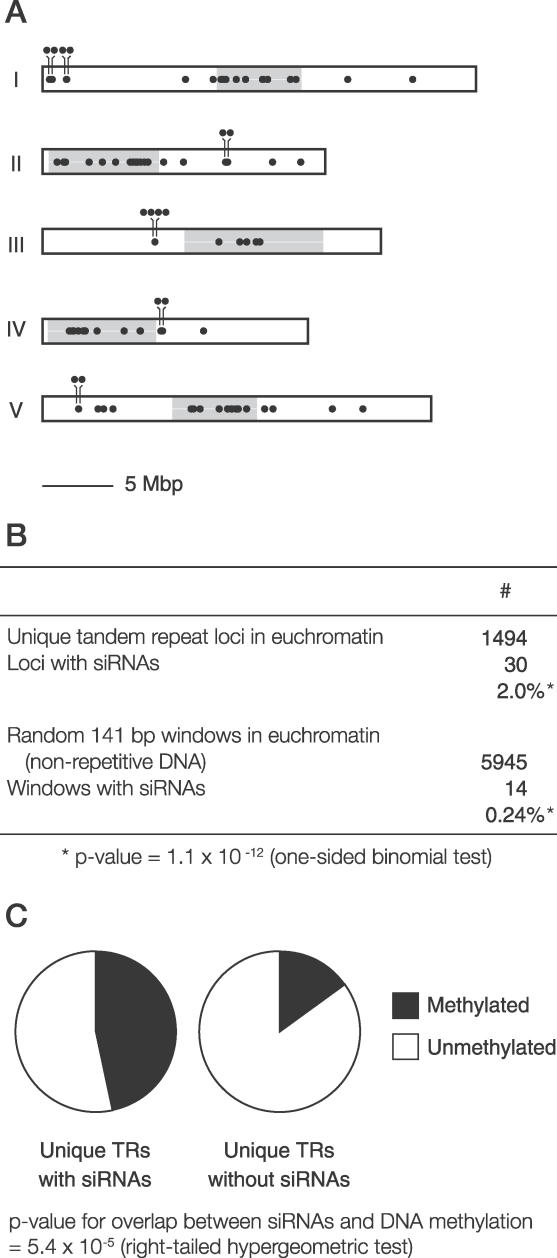
Association of siRNAs with Tandem Repeats throughout the *Arabidopsis* Genome (A) Chromosomal distribution of unique tandem repeats that produce siRNAs. Single-copy tandem repeats were identified (see text for details) and compared against a large database of cloned siRNAs [18]. The five *Arabidopsis* chromosomes are shown as rectangular boxes. Tandem repeats that overlap with dense or moderate siRNA clusters are shown as black dots (dots above the box represent euchromatic tandem repeats that were relatively close to each other). Pericentromeric heterochromatin is shown as gray shading. (B) Unique tandem repeats in euchromatin are enriched for siRNA production relative to randomly chosen sequences. Unique tandem repeats and a set of random sequences of similar size (141 bp is the median size of unique tandem repeats) were assessed for overlap with dense or moderate siRNA clusters. The probability of selecting 30/1494 siRNA-producing loci by chance (assuming the same siRNA-producing frequency as randomly chosen windows) was calculated with a one-sided binomial test. (C) DNA methylation of unique tandem repeats in euchromatin. Unique tandem repeats with and without siRNAs were compared to genome-wide DNA methylation data from an immunoprecipitation/tiling microarray experiment [37]. The fraction 221/1464 unique tandem repeats without siRNAs were methylated, compared with 14/30 unique tandem repeats that had siRNAs. The statistical significance of overlap between siRNA production and DNA methylation was calculated with a right-tailed hypergeometric test.

This preliminary analysis identified a set of 1494 “unique” tandem repeats from euchromatic regions of the *Arabidopsis* genome (Figure 6A), similar to the repeats found in *FWA*. We compared these to previously defined dense or moderate siRNA clusters, representing regions containing >11 cloned siRNAs without an intervening 500-bp gap [18] (single isolated siRNAs or sparse siRNA clusters were not considered in this study, because they might represent random matches). Thirty unique tandem repeats spread throughout the genome overlapped with siRNA clusters, representing 2.0% of unique tandem repeats. To compare unique tandem repeats with a relevant control sample of the *Arabidopsis* genome, we constructed a library of random 141-bp windows (the mean length of unique tandem repeats) from the euchromatic left arm of chromosome 1 using the same filtering procedure and threshold to remove repeated sequences or transposons. From a library of 5945 random windows, we found 14 that overlapped with dense or moderate siRNA clusters (0.24%). Thus, tandem repeats are nearly an order of magnitude more likely to produce siRNAs than random, nonrepeated sequences (Figure 6B). This enrichment is statistically significant, because the probability of selecting 30 siRNA-producing sequences at random was calculated at 1.0951 × 10^−12^ using a one-sided binomial distribution.

We next analyzed whether the presence of siRNAs was positively correlated with the presence of DNA methylation at unique tandem repeats, by comparing these loci to a genome-wide DNA methylation survey in which methylated DNA was immunoprecipitated and hybridized to *Arabidopsis* whole genome tiling microarrays (Figure 6C) [37]. Tandem repeats with siRNAs were methylated at a frequency of 46.7%, whereas only 15.1% of tandem repeats without siRNAs were methylated (similar to the genome average of 19%). This difference was highly statistically significant (Figure 6C). As tandem repeats with siRNAs are more frequently methylated than those without siRNAs, we infer that siRNA production may contribute to the maintenance of DNA methylation at these loci.

Because only a subset of unique tandem repeats produces siRNA, some other factors must determine whether stable siRNA production can exist at a given locus. We compared unique tandem repeats with and without siRNAs for several properties, including the overall length of the tandem repeat, repeat unit length, repeat copy number, average identity of repeat units, percentage of insertions and deletions (indels), and nucleotide sequence content. We found that tandem repeats with siRNAs were not significantly different in any of these characteristics (Figure S3). Thus, it may be that chromosomal context, some unknown feature of the DNA repeat sequence, or a particular set of chromatin modifications may influence which tandem repeats maintain siRNA production. We also analyzed the expression level of genes with unique tandem repeats in their promoters, and found that their expression was not significantly lower than those without tandem repeats (Figure S4). However, the importance of this result is unclear, because these tandem repeats may not affect promoter regions required for gene expression. Furthermore, tandem duplications in the genome may have been selected for those that do not repress important genes.

The euchromatic unique tandem repeats we analyzed represent only a small fraction of all tandem repeats that produce siRNAs. On a genome-wide scale, there is a strong correlation between tandem repeat character, siRNA production, and DNA methylation [4,18,37]; the vast majority of siRNA-producing tandem repeats are in pericentromeric heterochromatin, and are usually found at multiple genomic loci. Our results suggest that local repetitive nature is likely important in the initial recruitment of siRNA production to both unique and dispersed tandem repeats. Furthermore, continued siRNA production is correlated with the maintenance of DNA methylation. Thus, recognition of tandem repeats by RNA silencing proteins may be a general process for targeting gene silencing.

## Discussion

**Figure 7:**
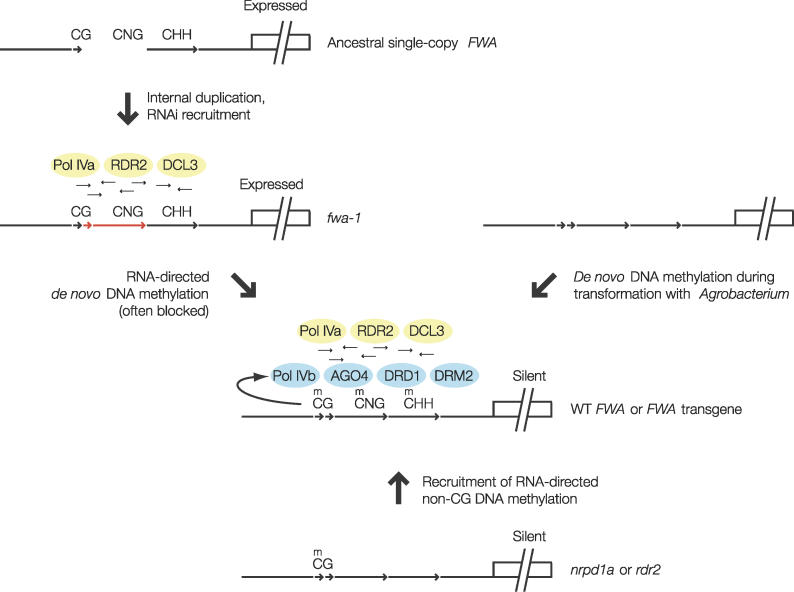
Model for RNA-Directed DNA Methylation at Tandem Repeats An ancestral single-copy gene may undergo internal duplication, creating tandem repeats that recruit siRNA-producing factors. After initial siRNA production, downstream RNA-directed DNA methylation may or may not occur efficiently at a particular locus, resulting in stable methylated or unmethylated genes, both of which produce siRNAs (small arrows). Transformed *FWA* efficiently recruits both siRNA production and downstream de novo DNA methylation. CG DNA methylation allows recruitment of siRNA-directed DNA methylation, providing a self-reinforcing feedback loop at silent loci.

Our results suggest a two-step model for de novo DNA methylation at tandem repeats (Figure 7). Most tandem repeats probably arise from single-copy sequences by internal duplication. The *FWA* tandem repeats were proposed to arise from a SINE transposon insertion [4]. However, plant SINE elements do not contain tandem repeats, and a secondary duplication event would therefore be necessary to create the current *FWA* 5′ untranslated region [38]. Additionally, the low level of similarity between the *FWA* tandem repeats and other *Arabidopsis* sequences (or any sequences in GenBank) suggests that other possibilities for their origin may be plausible. In particular, we propose a hypothetical yet parsimonious model in which the *FWA* tandem repeats could have arisen from an ancestral unrepeated sequence by a single internal duplication event (Figure 7). After their creation, tandem repeats appear to be preferential targets for siRNA-producing machinery, both at *FWA* and throughout the genome.

During the first step in which siRNAs are produced from tandem repeats, it seems likely that NRPD1a-containing Pol IV produces a single-stranded RNA that is a substrate for RDR2, which then produces a double-stranded RNA that is diced by DCL3. How NRPD1a-Pol IV might initially recognize a tandem repeat however is not known. Robust siRNA production can occur at this stage without the presence of DNA methylation, showing that RNAi proteins recognize the intrinsic properties of tandem repeats without features of silent chromatin. In the second step, downstream DNA methylation factors use siRNAs to cause DNA methylation. Our discovery that recruitment of siRNA production is separable from downstream RNA-directed DNA methylation is consistent with previously published data showing the existence of unmethylated *Arabidopsis* loci that produce siRNAs [30].

NRPD1b-containing Pol IV is not required to produce siRNAs, and we feel its role may be to generate a single-stranded transcript that is bound by an AGO4/siRNA complex, which in turn recruits the DRM2 DNA methyltransferase to a particular genomic locus [19,23]. DRD1 may remodel chromatin, assisting the activity of DRM2. This model for siRNA-directed DNA methylation is attractive, because it does not require the AGO4/siRNA complex to unwind the chromosomal double helix and interact with homologous single-stranded DNA. It is also consistent with evidence from Schizosaccharomyces pombe showing that transcription by RNA Pol II plays a role in RNAi-mediated transcriptional gene silencing [39,40].

A key insight from these studies is that the ability of siRNAs to cause DNA methylation depends on the nature of the siRNA-producing locus. siRNAs produced from methylated and unmethylated forms of *FWA* appear to be made by the same upstream RNAi components. However, siRNAs from unmethylated *fwa* do not target de novo DNA methylation in *cis,* and lack the ability to enhance silencing of transformed *FWA* in *trans*. One possible explanation is that these siRNAs may not be loaded into AGO4 after they are released from the DCL3 nuclease, or that factors downstream of AGO4 are not recruited to unmethylated loci.

In contrast to unmethylated loci, tandem repeats with pre-existing CG DNA methylation are susceptible to RNA-directed DNA methylation when siRNA production is recruited. This is shown by the fact that non-CG DNA methylation by NRPD1b/AGO4/DRD1/DRM2 returns to *FWA* and *MEA-ISR* when RNAi proteins are restored in a *nrpd1a* × *rdr2* cross (Figure 7). Exactly how CG DNA methylation facilitates RNA-directed DNA methylation is unclear. One possibility is that methyl-binding domain proteins interpret CG DNA methylation directly to recruit AGO4 and associated factors. Alternatively, another chromatin modification associated with DNA methylation could be key. The *Arabidopsis* genome contains many sites of CG-only DNA methylation, particularly in the coding region of genes [41]. One hypothesis that may explain why these sites do not recruit the non-CG methylation machinery is that CG-only sites lack tandem repeats and hence do not produce siRNAs. Although the maintenance methyltransferase enzyme MET1 is capable of maintaining CG-only regions of DNA methylation though a passive maintenance mechanism, tandem repeats that have both CG maintenance DNA methylation and persistent siRNA-directed non-CG DNA methylation may be more likely to retain their silent status over evolutionary time scales.

The process of plant transformation can also facilitate RNA-directed DNA methylation, because an *FWA* gene introduced by *Agrobacterium* is efficiently de novo methylated upon integration. Transformation also allows incoming *FWA* to cause de novo DNA methylation of the endogenous *FWA* gene (at a low frequency), bypassing the requirement for preexisting CG DNA methylation in recruiting RNA-directed DNA methylation. *Agrobacterium* introduces its T-DNA into plant cells as a single-stranded DNA molecule, which is copied into double-stranded DNA before its integration into the plant genome [42]. Multiple steps in this pathway could be responsible for potentiating siRNA-directed de novo DNA methylation. For example, increased siRNA levels could be a key factor associated with plant transformation. The effect of very high siRNA levels on de novo DNA methylation is shown by highly transcribed inverted repeat transgenes that target DNA methylation to homologous sequences independent of plant transformation [26]. Alternatively, a newly integrated transgene may possess a chromatin state that is permissive for RNA-directed DNA methylation, perhaps because of a particular set of histone modifications or lower nucleosome density. Such a permissive state might be maintained for several cell divisions after transformation (consistent with our observation that de novo DNA methylation occurs after fertilization of the female egg cell). A similarly susceptible state might occur for newly mobilized transposons.

Tandem repeats within a chromosome can increase their copy number by unequal crossing over or by gene conversion. Because these processes involve DNA breakage and rejoining reactions, they may act in the same way as *Agrobacterium* transformation to recruit molecular components that use siRNAs to cause de novo DNA methylation. In this way, the presence of siRNAs at unmethylated tandem repeats may potentiate DNA methylation during such expansions, or during periods of genome stress when chromatin is perturbed. Most tandem repeats are found at more than one genomic location and may be associated with transposons. In these cases, siRNA production may act as a kind of “immune memory” of dispersed repeats, allowing de novo DNA methylation to respond when selfish DNAs increase their copy number. The example of maize paramutation shows that tandem repeats in their natural context can indeed silence other genes in this manner [5].

Our findings also highlight the molecular mystery of how tandem repeats are initially recognized by RNAi proteins. Double-stranded RNAs that provide a Dicer substrate can be produced from inverted repeats by monodirectional transcription and fold-back, or from high-copy sequences by transcription of dispersed repeats in opposite directions. However, the mechanism that distinguishes unique tandem repeats from single copies of the same sequence remains unclear. One possibility is that NRPD1a-containing Pol IV and/or RDR2 may recognize unusual DNA or RNA structures that can only be made by tandem repeats. Regardless of the exact recognition mechanisms involved, the cellular RNAi machinery may be poised to produce siRNAs from newly created tandem repeats. In addition to providing a defense against potentially harmful sequences, this molecular mechanism may facilitate the evolution of genes whose normal expression is controlled by siRNAs and DNA methylation.

## Materials and Methods

### Plant materials.

Plants were grown under continuous light. All mutants have been previously described [10,11,22,24,26,29–31,33].

### *FWA* transformation and flowering time analysis.

*FWA* transformation was performed as described [11]. For heterozygous mutants experiments, early- and late-flowering time was determined by comparison to isogenic ecotypes (Figure S1).

### *FWA* deletion analysis.

Unless noted, *FWA* deletions were within the context of the XbaI-HindIII fragment from −2124 to 3876 (where the start codon is at +1) and were cloned into pCAMBIA1300. An *FWA* transgene where a BglII site is changed to EcoRI has been described [27]. The “repeats deleted” transgene lacks −1064 to −564. The “single-copy” transgene lacks −1026 to −779. The *FWA* promoter lacking the open reading frame contains nucleotides −2124 to 0, placed before a *GFP-GUS* open reading frame and cloned into pCAMBIA1300 from XbaI to HindIII (*GFP-GUS* was derived from pBGWFS7) [43]. The “repeats only” construct includes nucleotides −1072 to −559, placed before *GFP-GUS* and cloned into pCAMBIA1300.

### Bisulfite sequencing and bisulfite PCR/restriction enzyme digestion assays.

Bisulfite sequencing was performed as described [14,28]. For “single-copy” *FWA,* the region analyzed had the same boundaries as full-length *FWA* (nucleotides −1055 to −608). Bisulfite sequencing data are summarized in Table S1. PCR primer sequences for bisulfite sequencing and bisulfite PCR/restriction digest assays are listed in Table S2.

### Northern blot analysis of small RNAs.

Northern blots were performed as described [30]. The sequences of the *FWA* LNA probes used in this study are listed in Table S2.

### RT-qPCR analysis of gene expression.

RNA was extracted from rosette leaves with TRIzol reagent (Invitrogen, Carlsbad, California, United States), DNAse treated with the DNA-free kit (Ambion, Austin, Texas, United States), and converted to cDNA with SuperScript II reverse transcriptase (Invitrogen) using a poly-T primer. *FWA* and *ACT7* PCR primer sequences are listed in Table S2.

## Supplementary Information

Figure S1 Flowering-Time Distribution for *rdr2–1, ago4–1,* and *drm1–1 drm2–1* Compared to Their Respective Wild-Type Ecotypes (126 KB PDF)

Figure S2 *FWA* siRNAs Are Present when the *FWA* Gene Is Unmethylated (558 KB PDF)

Figure S3 Properties of Unique Tandem Repeats with and without siRNAs (233 KB PDF)

Figure S4 Expression Level of Genes with and without Unique Tandem Repeats in Their Promoter Regions (86 KB PDF)

Table S1 Summary of Bisulfite Genomic Sequencing Data (79 KB PDF)

Table S2 Sequences of Oligonucleotides Used in this Study (26 KB PDF)

## Abbreviations

LNA: locked nucleic acid
RNAi: RNA interference
siRNA: small interfering RNA
