# Supplementary material for: Two-Step Recruitment of RNA-Directed DNA Methylation to Tandem Repeats
Source: PLoS Biol. 2006 Oct 24;4(11):e363. doi: 10.1371/journal.pbio.0040363 (PMC1618421; doi:10.1371/journal.pbio.0040363)
Supplement: Supplementary file 1 [file pbio.0040363.sg001.pdf]

Supplementary Material for “Two-Step Recruitment of RNA-directed DNA Methylation to Tandem Repeat Sequences.” by Simon W.-L. Chan, Yana Bernatavichute, Xiaoyu Zhang and Steven E. Jacobsen.

Supplementary Figure S1. Flowering time distribution for *rdr2-1*, *ago4-1*, and *drm1-1 drm2-1* compared to their respective wild-type ecotypes.

Histograms show rosette leaf number on the X axis and number of plants on the Y axis. Late flowering was defined as 23 or more leaves for *rdr2-1/RDR2*, 10 or more leaves for *ago4-1/AGO4*, and 12 or more leaves for *drm1-1/DRM1 drm2-1/DRM2*.

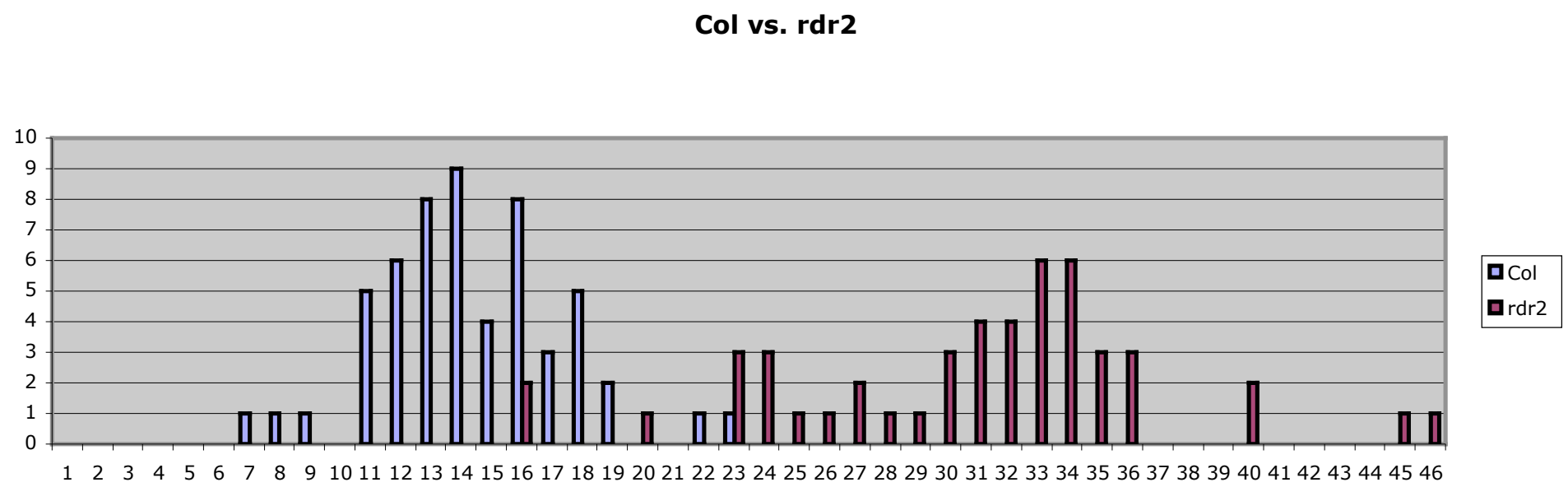

Number of plants

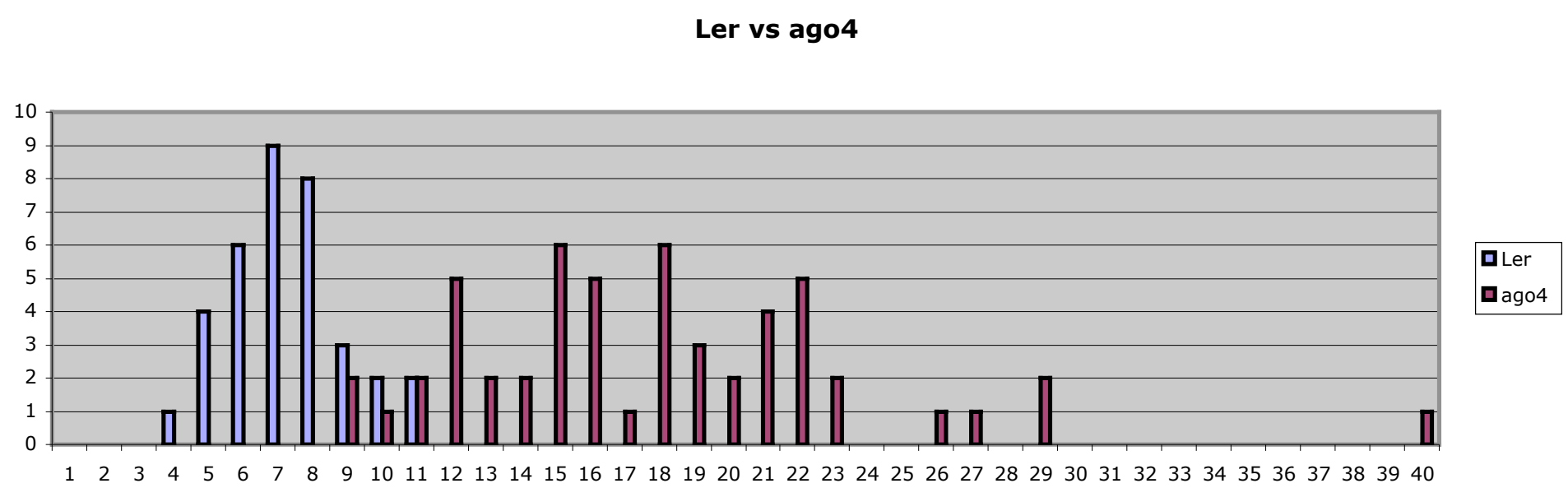

Number of plants

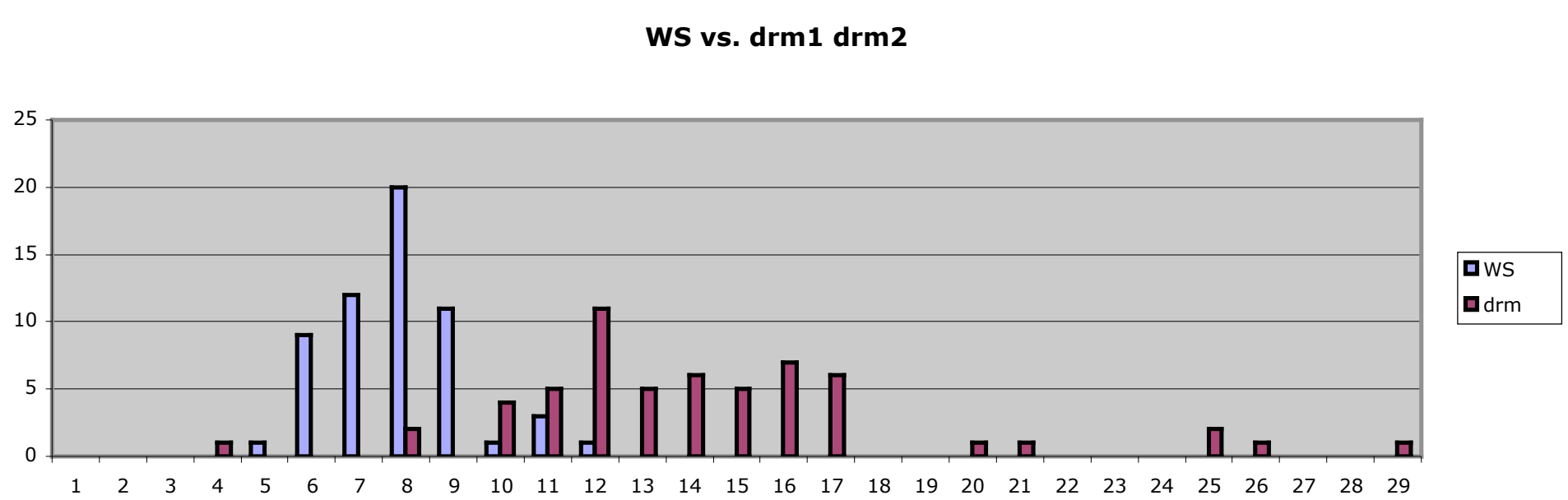

Number of plants

Number of rosette leaves
