# Supplementary material for: Two-Step Recruitment of RNA-Directed DNA Methylation to Tandem Repeats
Source: PLoS Biol. 2006 Oct 24;4(11):e363. doi: 10.1371/journal.pbio.0040363 (PMC1618421; doi:10.1371/journal.pbio.0040363)
Supplement: Supplementary file 2 [file pbio.0040363.sg002.pdf]

Supplementary Figure S2

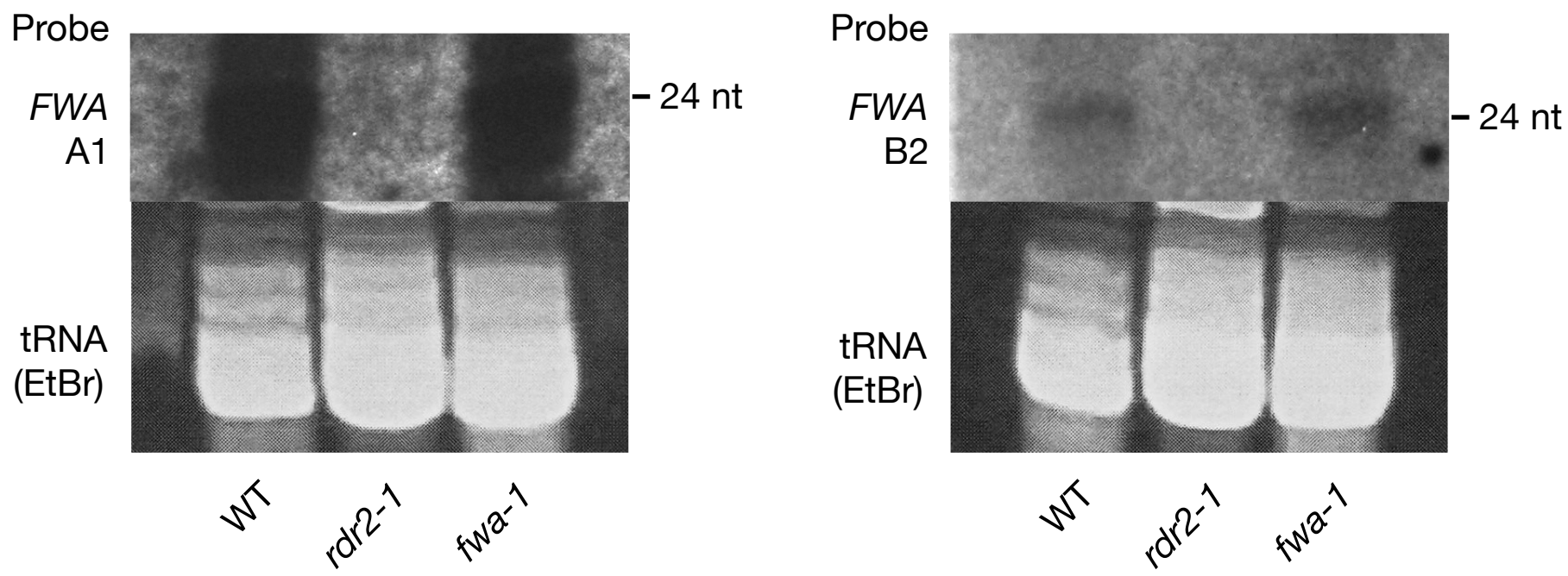

siRNAs from the indicated genotypes were probed with LNA probes corresponding to the *FWA* tandem repeats.

The *FWA* tandem repeats are base pairs -1063 to -570. The probe used in Figures 4 and 5 is antisense -889 to -936 (within first large repeat).

Probe A1 is antisense -1026 to -1063 (within small repeat).
Probe B2 is antisense -602 to -643 (within second large repeat).
