# Supplementary material for: Two-Step Recruitment of RNA-Directed DNA Methylation to Tandem Repeats
Source: PLoS Biol. 2006 Oct 24;4(11):e363. doi: 10.1371/journal.pbio.0040363 (PMC1618421; doi:10.1371/journal.pbio.0040363)
Supplement: Supplementary file 3 [file pbio.0040363.sg003.pdf]

Supplemental Figure 3. Comparisons of the repeat characteristics of all TRs, unique TRs and unique TRs associated with siRNAs

all TRs

all unique TRs

unique TRs

with siRNAs

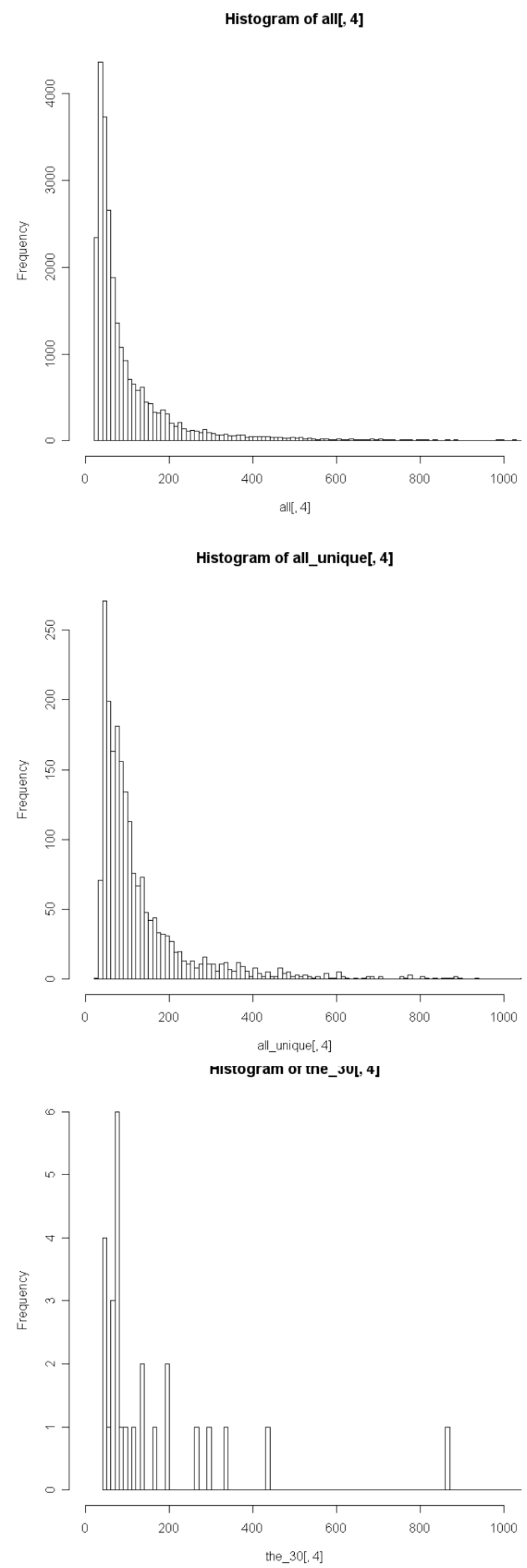

Supplemental Figure 3A. Comparison of the length of all TRs (top), unique TRs (middle) and unique TRs associated with siRNAs (bottom).

x-axis: length of each TR

y-axis: frequency

all TRs

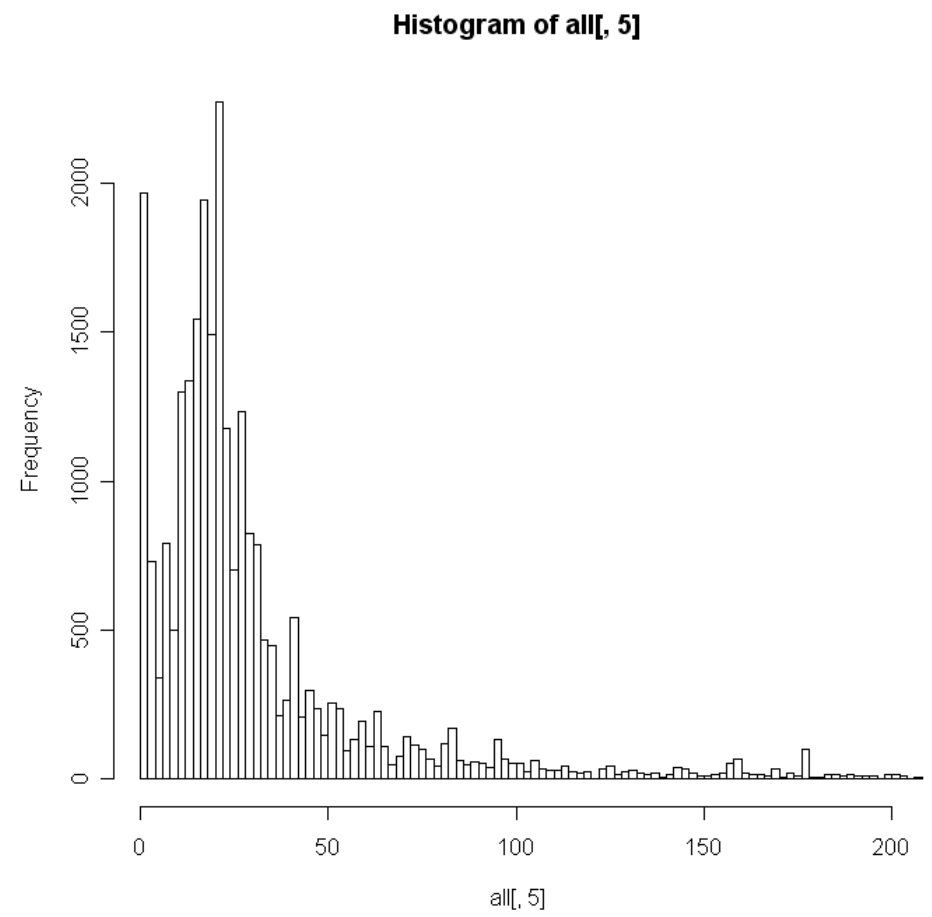

all unique TRs

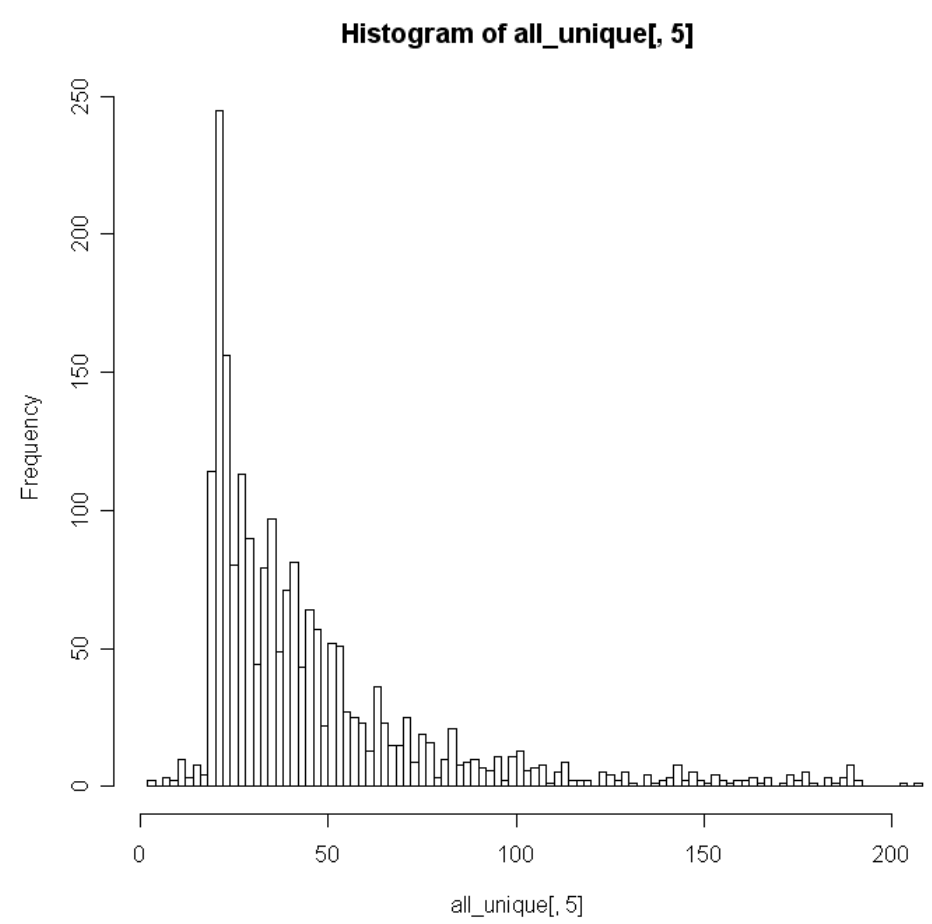

unique TRs

with siRNAs

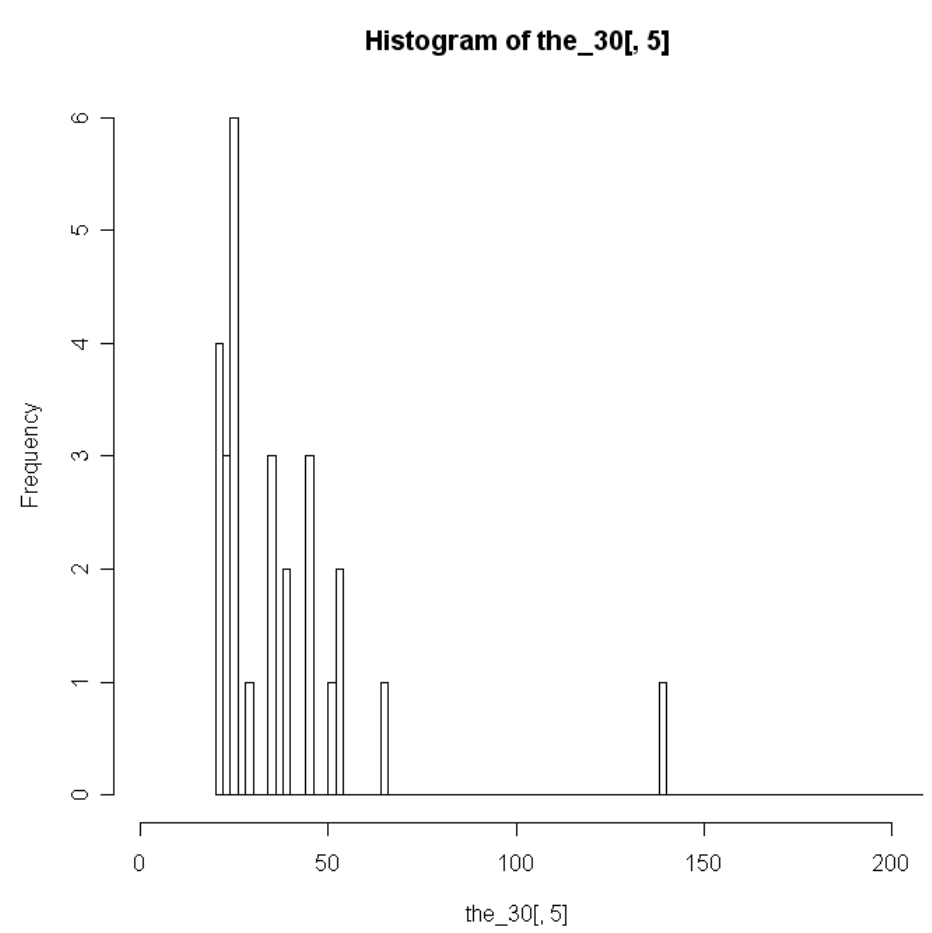

Supplemental Figure 3B. Comparison of the repeat unit length of all TRs (top), unique TRs (middle) and unique TRs associated with siRNAs (bottom).

x-axis: repeat unit length length of each TR

y-axis: frequency

all TRs

all unique TRs

unique TRs

with siRNAs

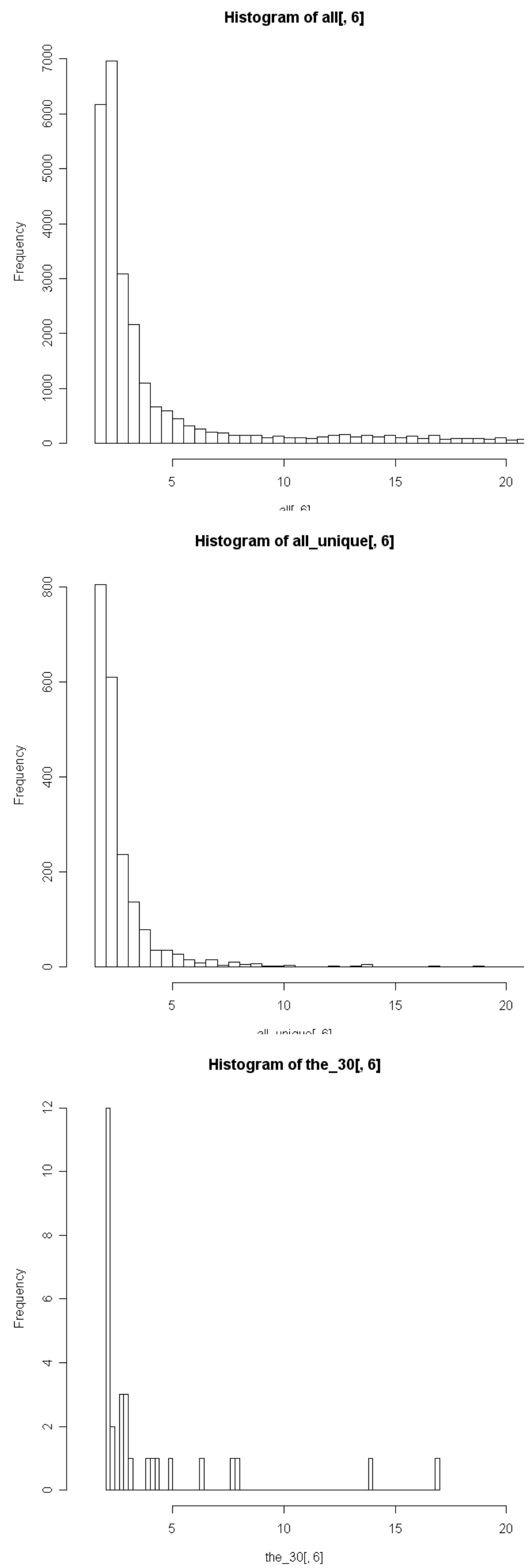

Supplemental Figure 3C. Comparison of the number of repeat units within each TR, for all TRs (top), unique TRs (middle) and unique TRs associated with siRNAs (bottom).

x-axis: number of repeat units within each TR

y-axis: frequency

all TRs

all unique TRs

unique TRs

with siRNAs

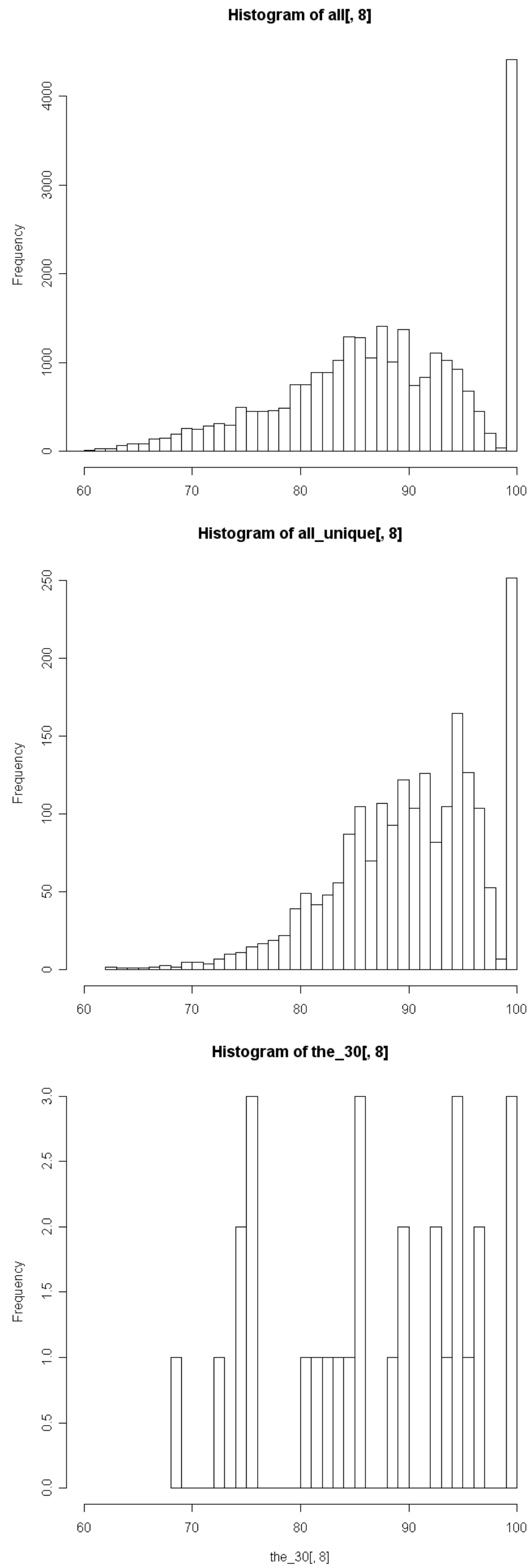

Supplemental Figure 3D. Comparison of the average identity among the repeat units within each TR, for all TRs (top), unique TRs (middle) and unique TRs associated with siRNAs (bottom).

x-axis: average identity among the repeat units within each TR

y-axis: frequency

all TRs

all unique TRs

unique TRs

with siRNAs

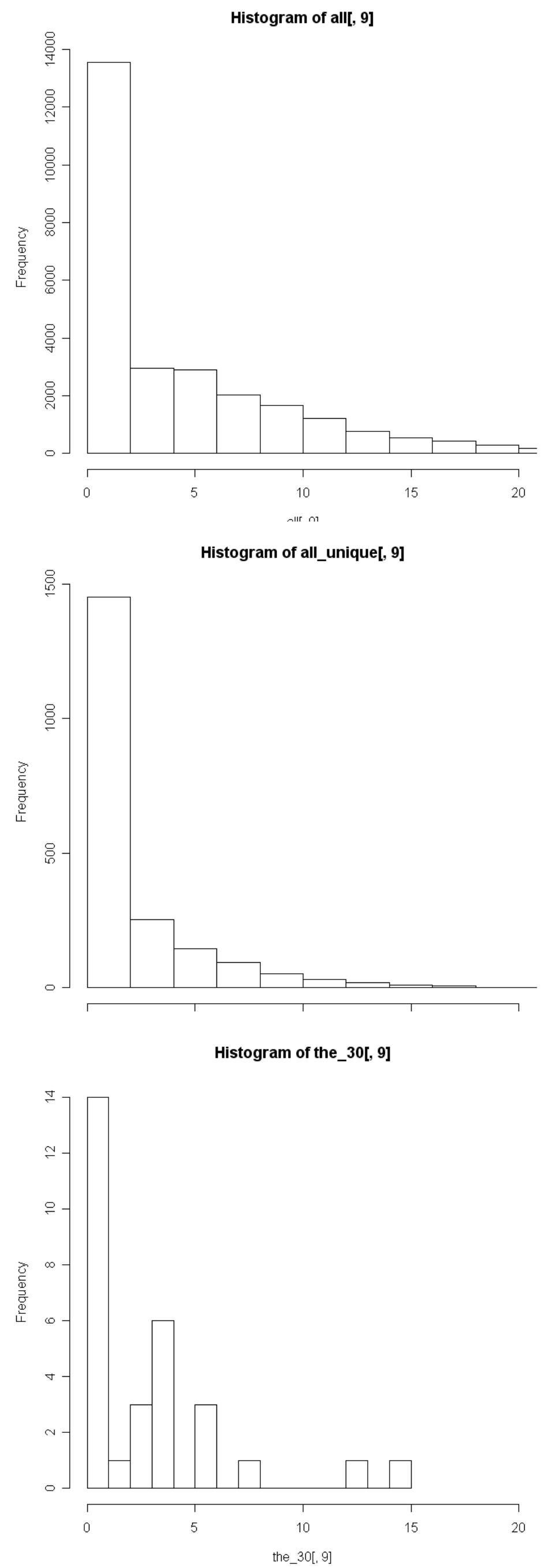

Supplemental Figure 3E. Comparison of the percentage of insertions and deletions (indels) among the repeat units within each TR, for all TRs (top), unique TRs (middle) and unique TRs associated with siRNAs (bottom).

x-axis: percentage of indels among the repeat units within each TR

y-axis: frequency

all TRs

all unique TRs

unique TRs

with siRNAs

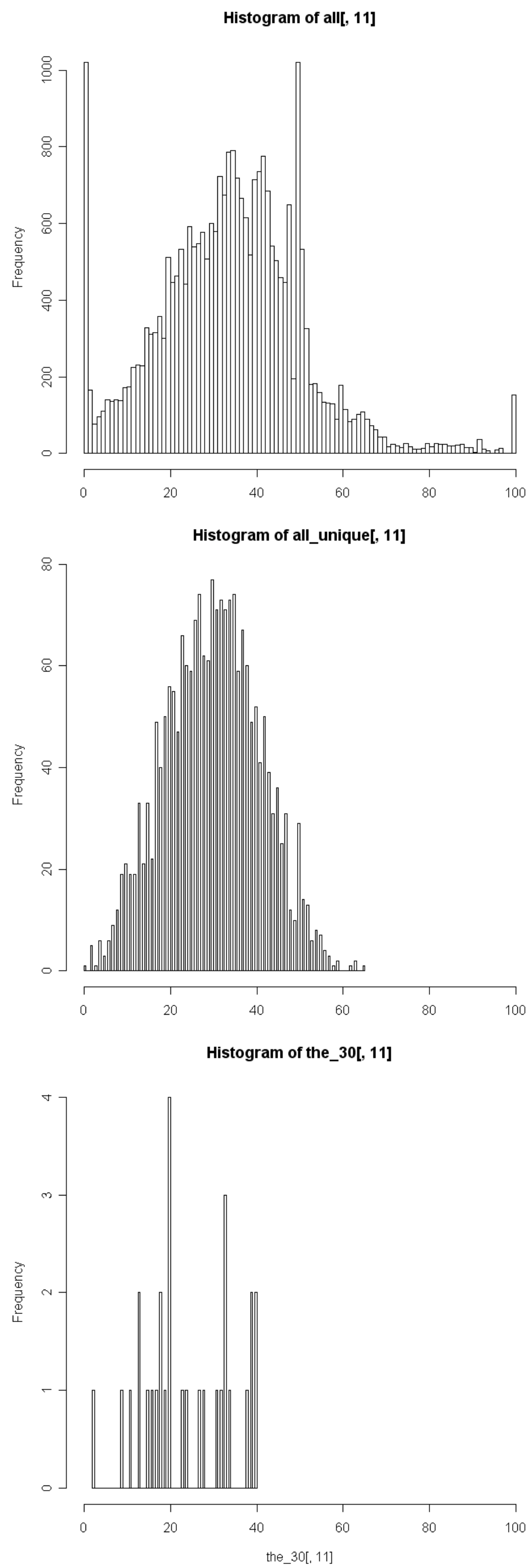

Supplemental Figure 3F. Comparison of the sequence content (% of A) of all TRs (top), unique TRs (middle) and unique TRs associated with siRNAs (bottom).

x-axis: percentage of indels among the repeat units within each TR

y-axis: frequency

all TRs

all unique TRs

unique TRs

with siRNAs

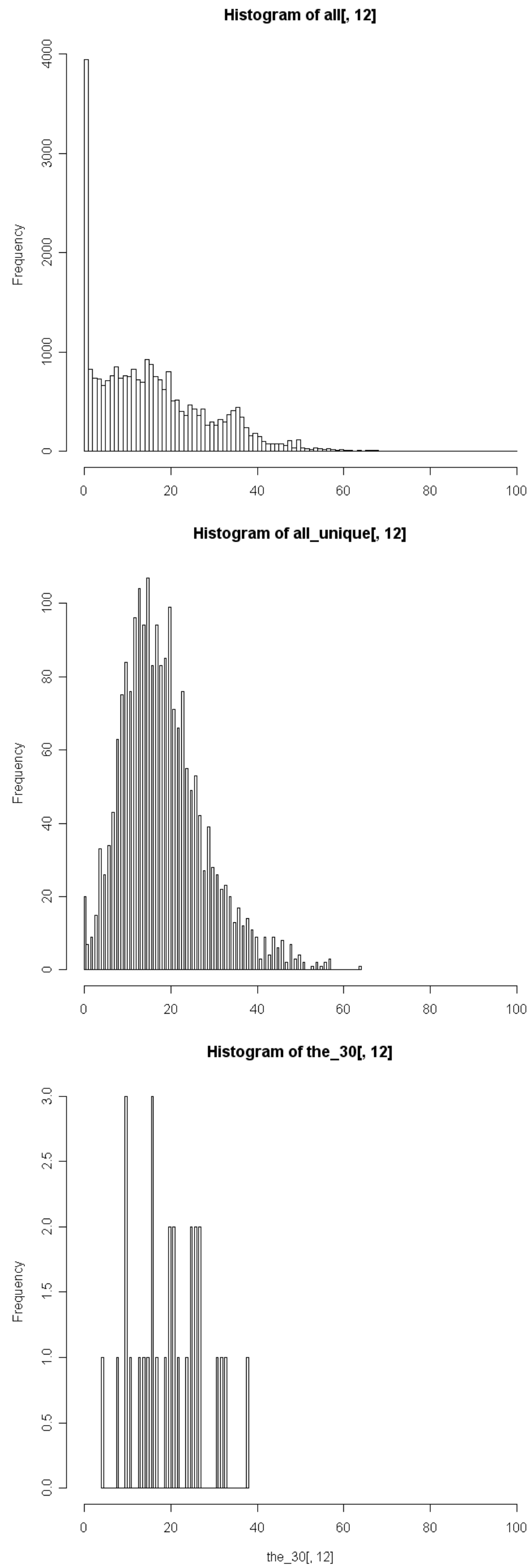

Supplemental Figure 3G. Comparison of the sequence content (% of C) of all TRs (top), unique TRs (middle) and unique TRs associated with siRNAs (bottom).

x-axis: percentage of indels among the repeat units within each TR

y-axis: frequency

all TRs

all unique TRs

unique TRs

with siRNAs

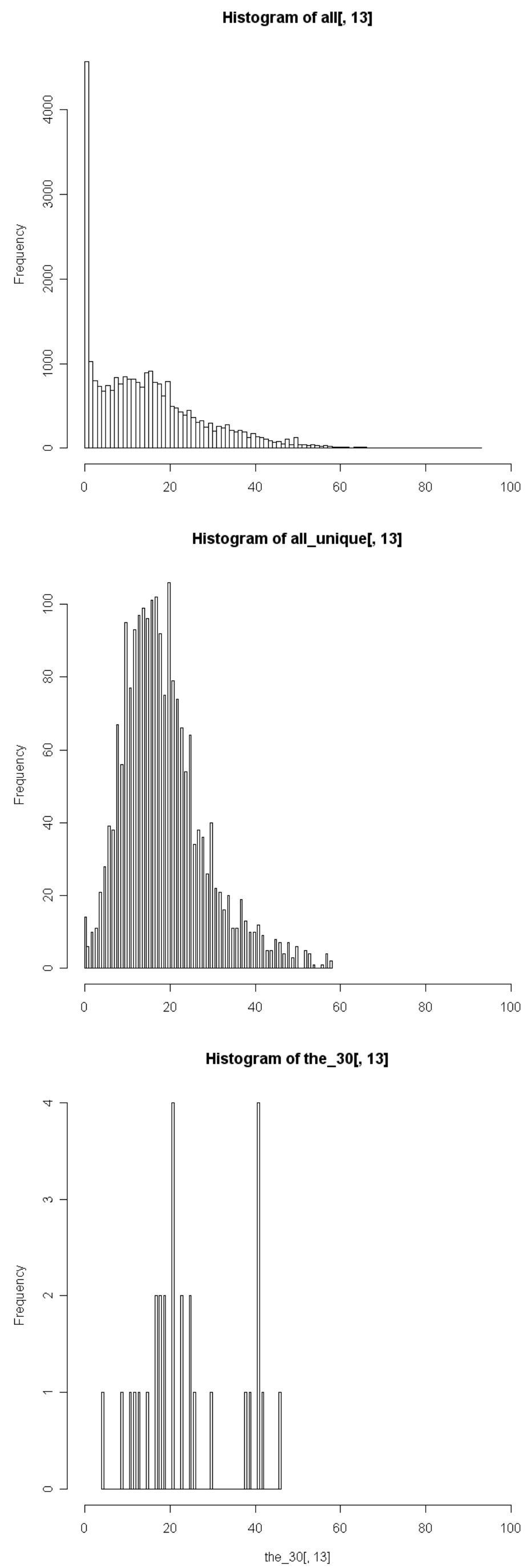

Supplemental Figure 3H. Comparison of the sequence content (% of G) of all TRs (top), unique TRs (middle) and unique TRs associated with siRNAs (bottom).

x-axis: percentage of indels among the repeat units within each TR

y-axis: frequency

all TRs

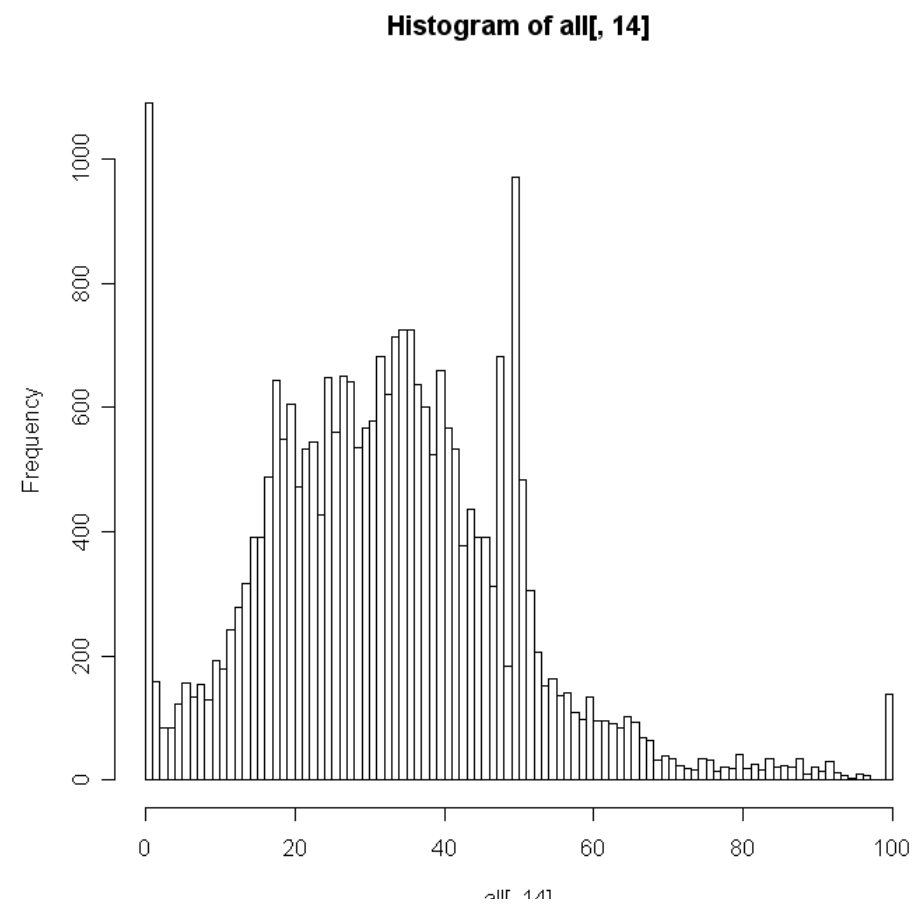

all unique TRs

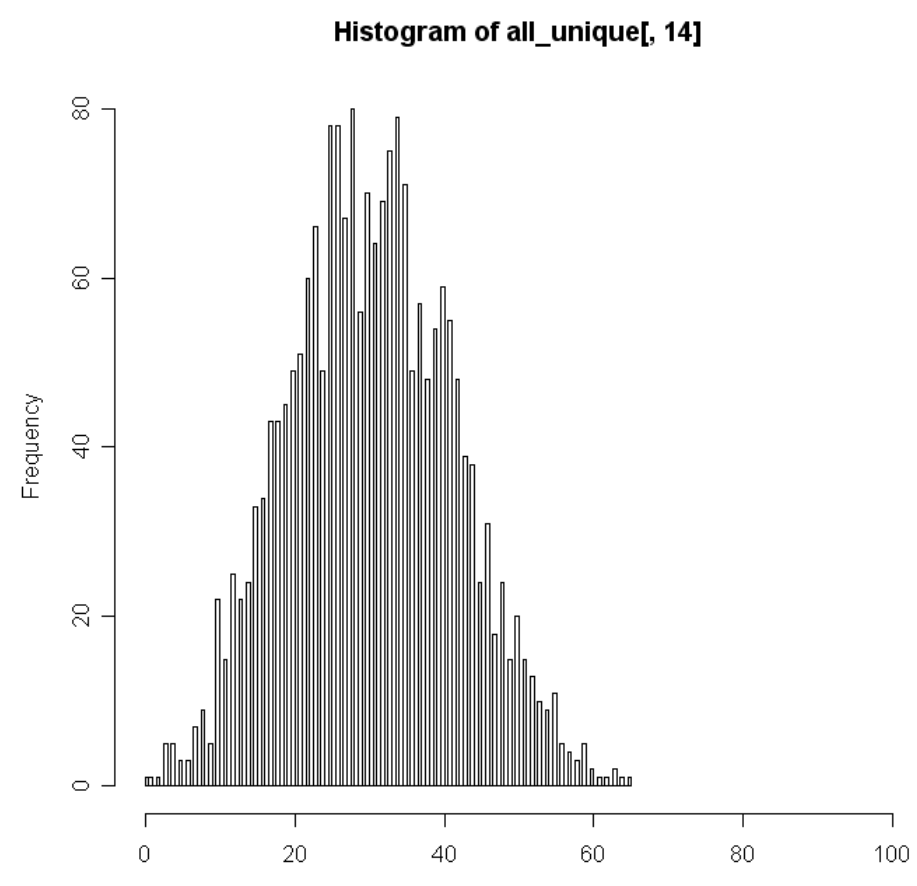

unique TRs

with siRNAs

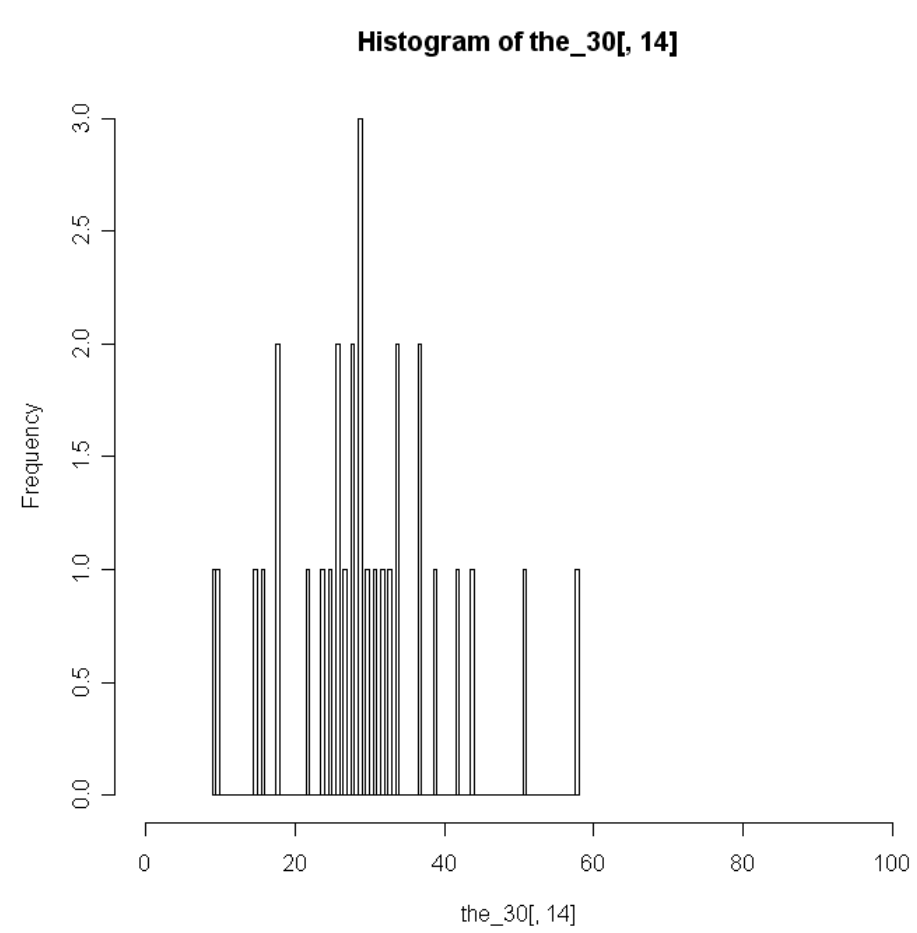

Supplemental Figure 3I. Comparison of the sequence content (% of T) of all TRs (top), unique TRs (middle) and unique TRs associated with siRNAs (bottom).

x-axis: percentage of indels among the repeat units within each TR

y-axis: frequency
