# Supplementary material for: Two-Step Recruitment of RNA-Directed DNA Methylation to Tandem Repeats
Source: PLoS Biol. 2006 Oct 24;4(11):e363. doi: 10.1371/journal.pbio.0040363 (PMC1618421; doi:10.1371/journal.pbio.0040363)
Supplement: Supplementary file 4 [file pbio.0040363.sg004.pdf]

Supplementary Figure S4

Expression level of genes with and without unique tandem repeats in their promoter regions.

The distribution of expression levels of A) all genes, B) genes containing unique tandem repeats (TRs, see text for a detailed definition of unique tandem repeats) within their promoter regions (defined as 1 kb upstream of transcription start site), and C) genes containing unique tandem repeats within their promoter regions that are also associated with siRNAs. The expression level of each gene is the average over 79 tissues and conditions (Schmid et al. 2005 Nat Genet 37: 501-506) shown in log2 scale.

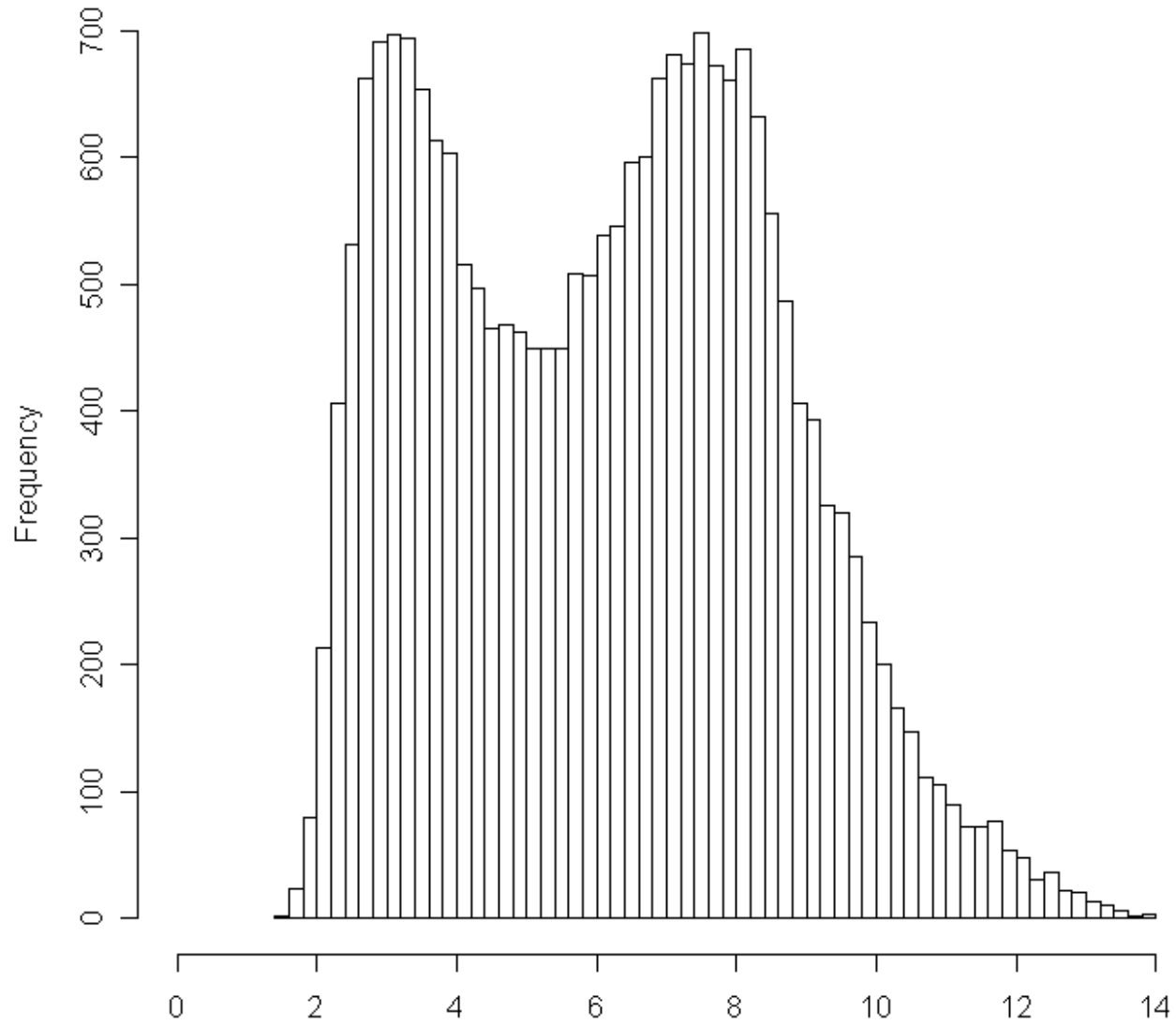

A) All genes

- x: intensity (log2 of RMA mean)
- y: frequency

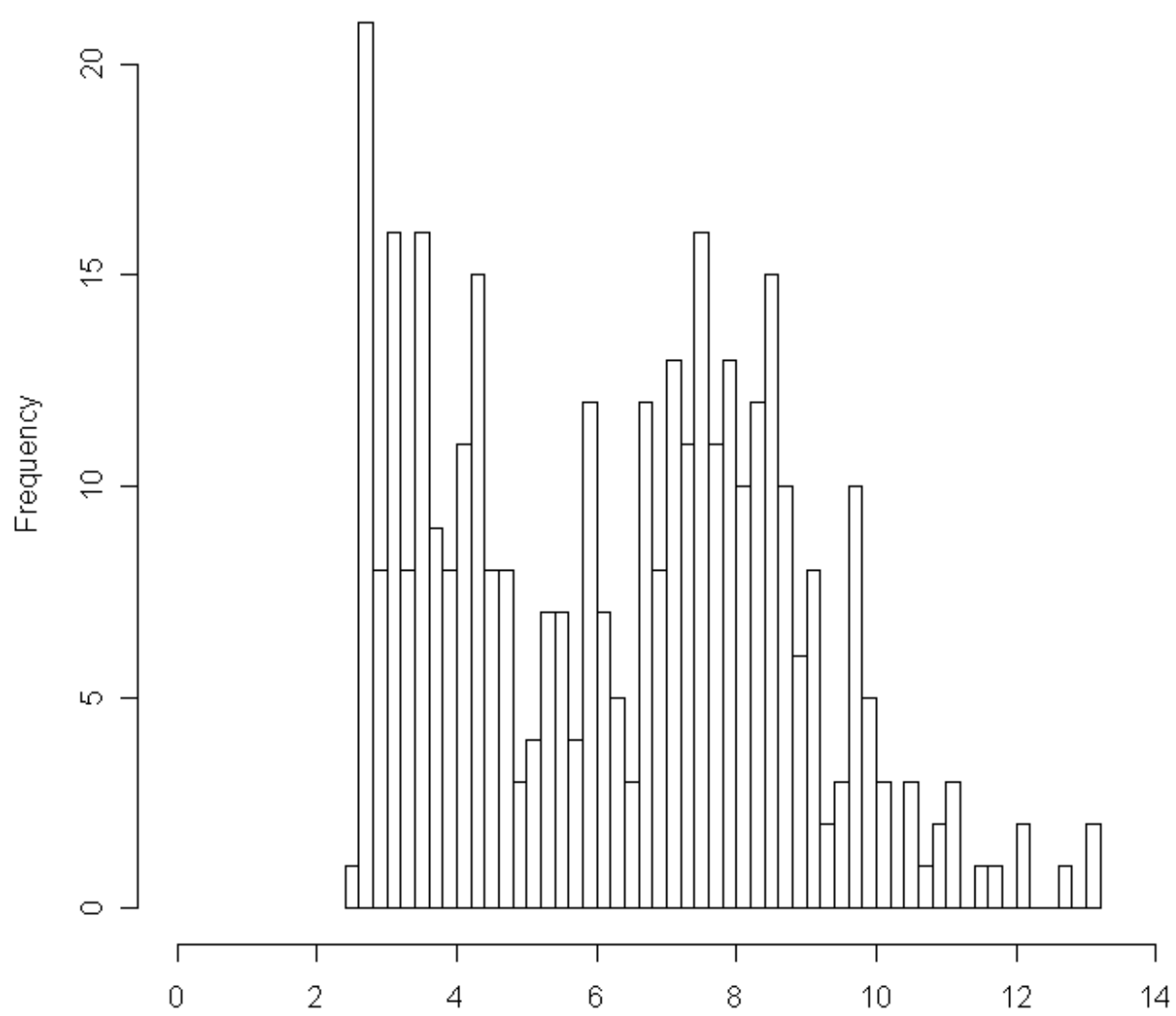

B) Genes with TRs in promoters (1 kb)

- x: intensity (log2 of RMA mean)
- y: frequency

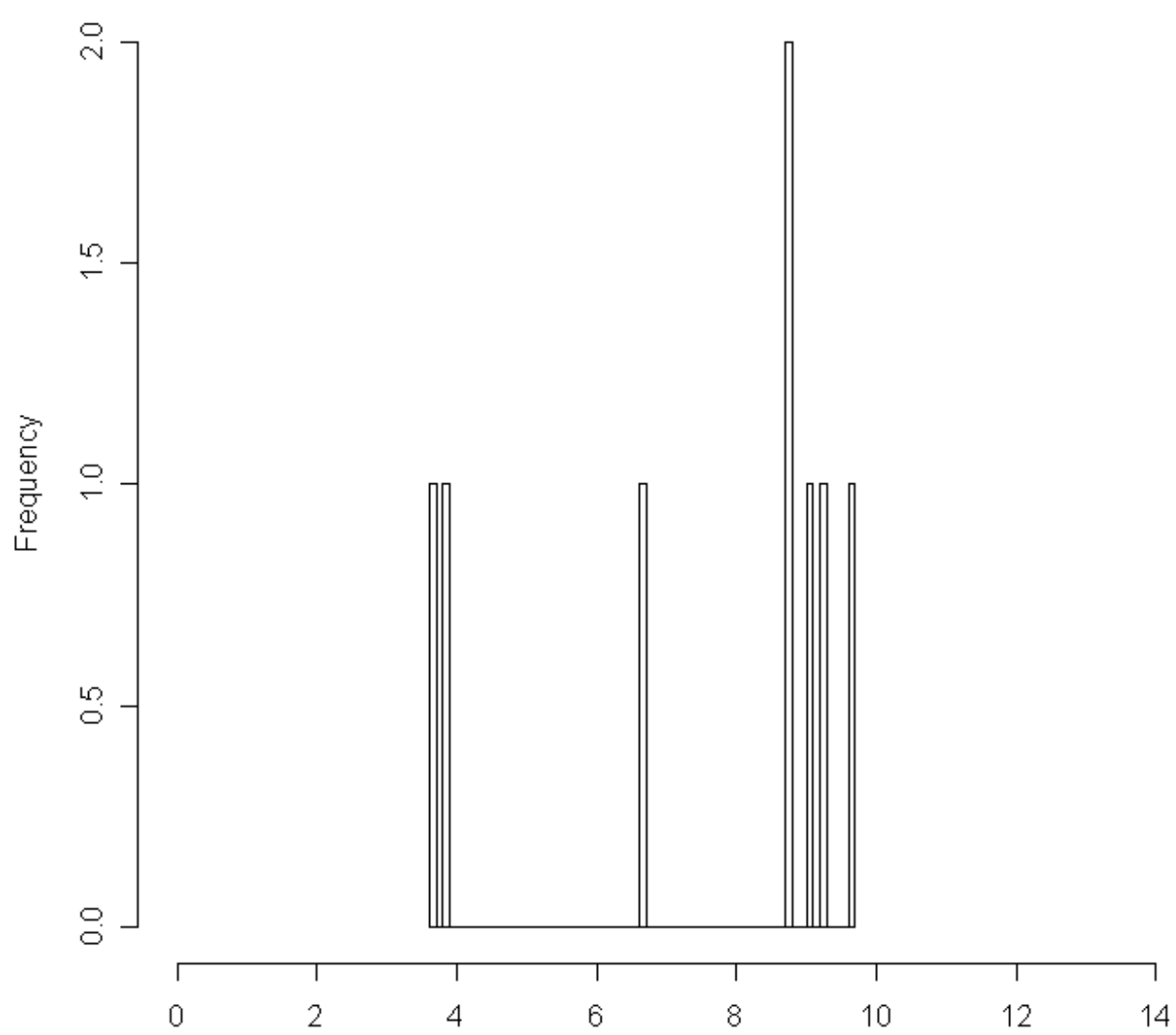

C) Genes with siRNA-associated TRs in promoters (1 kb)

- x: intensity (log2 of RMA mean)
- y: frequency
