# Supplementary material for: Two-Step Recruitment of RNA-Directed DNA Methylation to Tandem Repeats
Source: PLoS Biol. 2006 Oct 24;4(11):e363. doi: 10.1371/journal.pbio.0040363 (PMC1618421; doi:10.1371/journal.pbio.0040363)
Supplement: Supplementary file 5 [file pbio.0040363.st001.pdf]

Supplementary Material for "Two-Step Recruitment of RNA-directed DNA Methylation to Tandem Repeat Sequences." by Simon W.-L. Chan, Yana Bernatavichute, Xiaoyu Zhang and Steven E. Jacobsen.

Table S1. Bisulfite genomic sequencing data.

Number of cytosines methylated in different sequence contexts in cloned PCR products from bisulfite-treated DNA.

FWA T1 transgene. 20 CG sites, 10 CNG sites, 61 asymmetric sites.

FWA "single copy" T1 transgene. 11 CG sites, 5 CNG sites, 26 asymmetric sites.

	CG methylated	CNG methylated	CHH (asymmetric) methylated
Col + *FWA*	117/240 (48.8%)	32/120 (26.7%)	84/732 (11.5%)
Col + *FWA* single copy	0/286 (0%)	0/130 (0%)	1/676 (0.15%)
fwa-1 + *FWA* late flowering T1	0/420 (0%)	0/210 (0%)	5/1281 (0.4%)

FWA endogene. 20 CG sites, 10 CNG sites, 61 asymmetric sites.

	CG methylated	CNG methylated	CHH (asymmetric) methylated
L*er*	335/400 (84%)	31/200 (16%)	83/1220 (6.8%)
rdr2-1	352/400 (88%)	0/200 (0%)	6/1220 (0.5%)
nrpd1a-1	347/400 (87%)	7/200 (3.5%)	10/1220 (0.8%)
F1 *rdr2-1 x nrpd1a-1*	181/240 (75%)	13/120 (11 %)	53/752 (7%)
kyp-2	375/440 (85.2%)	20/242 (8.3%)	51/1342 (3.8%)
F1 *kyp2 rdr2-1 x kyp2 nrpd1a-1*	297/340 (87.3%)	21/170 (12.4%)	94/1037 (9.1%)
