# Supplementary material for: Two-Step Recruitment of RNA-Directed DNA Methylation to Tandem Repeats
Source: PLoS Biol. 2006 Oct 24;4(11):e363. doi: 10.1371/journal.pbio.0040363 (PMC1618421; doi:10.1371/journal.pbio.0040363)
Supplement: Supplementary file 6 [file pbio.0040363.st002.pdf]

Supplementary Table S2
Chan et al "Two-step recruitment of RNA-directed DNA methylation to tandem repeats"

PCR primer sequence	Purpose
TGACTGACAGCTGAAAATGGGATGTGGAT	ago4-1 genotyping (CAPS marker with AvaII)
GCCACTCCCTAGAACTCACCACCTAAGTT	ago4-1 genotyping
tag cat ctg aat ttc ata acc aat ctc gat aca c	rdr2-1 SAIL T-DNA left border primer
aca cat tag gac taa caa att tac c	rdr2-1 genotyping
atg gtg tca gag acg acg acg aac cga tca	rdr2-1 genotyping
ggttttatattaatattaaagagttatgggtygaagttt	bisulfite sequencing of FWA
caaaatactttacacataaacraaaaacaaacaaatcraa	bisulfite sequencing of FWA
caaaatactttacacataaacraaaaacaaacaaatcraa	bisulfite PCR/ClaI assay of FWA methylation
GTttAAGTGtTATTTGGTTGTTTAAGGTTGtTTTTAGtAt	bisulfite PCR/ClaI assay of FWA methylation
ag+caa+cct+taa+aca+acc+aaa+tag+cac+ttg+gac+caa+tgg+cga+a	FWA LNA probe from Figure 4A
AT+ATG+AGA+TTC+TCG+ACG+GAA+AGA+TGT+ATG+GGC+TTCG	FWA LNA probe A1 (Supplementary Figure S3)
CTG+ATT+GTC+AGT+ATCCT+ACA+AATC+GAT+AAATC+GTA+AGA+AG	FWA LNA probe B2 (Supplementary Figure S3)
	(+ indicates LNA at following position)
CCTTCGGGATTTTCGATAGTGCCA	Taqman probe for FWA real-time RT-qPCR
CCCACCAAGATCTGAAGTCC	FWA RT-qPCR
CAGGTGCAATGGTGGTGTAT	FWA RT-qPCR
Ttt tcc cta gtt gag atg gga att gaa	Taqman probe for ACT7 real-time RT-qPCR
Cagcatcatcacaagcatcc	ACT7 RT-qPCR
Tcgtggtggtgagtttgttac	ACT7 RT-qPCR
aaa gtg gtt gta gtt tat gaa agg ttt tat	bisulfite PCR/BamHI assay of MEA-ISR methylation
CTTAAAAAATTTTCAACTCATTTTTAAAAAA	bisulfite PCR/BamHI assay of MEA-ISR methylation
